# High-throughput and high-accuracy single-cell RNA isoform analysis using PacBio circular consensus sequencing

**DOI:** 10.1038/s41467-023-38324-9

**Published:** 2023-05-06

**Authors:** Zhuo-Xing Shi, Zhi-Chao Chen, Jia-Yong Zhong, Kun-Hua Hu, Ying-Feng Zheng, Ying Chen, Shang-Qian Xie, Xiao-Chen Bo, Feng Luo, Chong Tang, Chuan-Le Xiao, Yi-Zhi Liu

**Affiliations:** 1 State Key Laboratory of Ophthalmology, Zhongshan Ophthalmic Center, Sun Yat-sen University, Guangdong Provincial Key Laboratory of Ophthalmology and Visual Science, Guangzhou, 510060 China; 2 College of Life Sciences, University of Chinese Academy of Sciences, Beijing, 100049 China; 3 Guangdong Key Laboratory of Liver Disease Research, the Third Affiliated Hospital of Sun Yat-sen University, Guangzhou, 510630 China; 4 Key Laboratory of Genetics and Germplasm Innovation of Tropical Special Forest Trees and Ornamental Plants, Ministry of Education, College of Forestry, Hainan University, Haikou, 570228 China; 5 Beijing Institute of Radiation Medicine, Beijing, China; 6 School of Computing, Clemson University, Clemson, SC 29634-0974 USA; 7 BGI Genomics, BGI Shenzhen, Shenzhen, China; 8 Research Unit of Ocular Development and Regeneration, Chinese Academy of Medical Sciences, Beijing, China

**Keywords:** RNA sequencing, Transcriptomics, Genome-wide analysis of gene expression, High-throughput screening, Next-generation sequencing

## Abstract

Although long-read single-cell RNA isoform sequencing (scISO-Seq) can reveal alternative RNA splicing in individual cells, it suffers from a low read throughput. Here, we introduce HIT-scISOseq, a method that removes most artifact cDNAs and concatenates multiple cDNAs for PacBio circular consensus sequencing (CCS) to achieve high-throughput and high-accuracy single-cell RNA isoform sequencing. HIT-scISOseq can yield >10 million high-accuracy long-reads in a single PacBio Sequel II SMRT Cell 8M. We also report the development of scISA-Tools that demultiplex HIT-scISOseq concatenated reads into single-cell cDNA reads with >99.99% accuracy and specificity. We apply HIT-scISOseq to characterize the transcriptomes of 3375 corneal limbus cells and reveal cell-type-specific isoform expression in them. HIT-scISOseq is a high-throughput, high-accuracy, technically accessible method and it can accelerate the burgeoning field of long-read single-cell transcriptomics.

## Abstract (web summary)

Long-read single-cell RNA isoform sequencing can elucidate the intricate landscape of alternative RNA splicing in individual cells, but it suffers from a low read throughput. Here, the authors develop circular consensus sequencing methods to allow high-throughput and high-accuracy single-cell RNA isoform sequencing.

## Introduction

Single-cell RNA sequencing (scRNA-Seq) technologies can resolve expression heterogeneity across different cell types and states and have been widely used in fields involving complex biological and pathological processes, such as developmental biology, oncology, neuroscience, and immunology^1–5^. While next-generation sequencing (NGS) based high-throughput scRNA-Seq^6^ technologies using cell barcoding strategies have low sequencing error rates and are cost-effective, they are more powerful in gene expression quantification than resolving complex RNA isoforms^7^. Recently, through combining single-molecule long-read sequencing technology (PacBio or Oxford Nanopore sequencing), researchers have developed multiple microfluidics^8–13^ and well^14,15^ based single-cell isoform RNA-Seq (ScISOr-Seq^16^) approaches. Long-read single-cell isoform RNA-Seq enables comprehensive study of single-cell alternative splicing and fusion transcripts^11,16^. It also has the potential to learn special characteristics of RNA poly(A) tails such as length control principals^17^ and non-adenosine residues^18^.

However, existing long-read single-cell isoform RNA-Seq methods suffer from a low read throughput for two reasons. First, the 10× Genomics single-cell preparation pipeline of ScISOr-Seq introduces a high proportion (~50%) of undesirable cell-barcode-free reads, mostly template-switching oligonucleotide (TSO) artifacts formed during library construction^12^. These artifacts result in a waste of ~50% of sequencing resources^12,19^. Second, the long-read sequencing technologies (Nanopore and PacBio) have their respective limits. Although the Nanopore PromethION platform can generate >100 million raw reads per flow cell, its cell-barcode demultiplex efficiency is low due to the relatively high read error rate. PacBio’s Sequel II platform CCS mode recommends a 10–20 kb library insert size for achieving both high read quality and good throughput^20^. However, an ordinary cDNA library usually has a much shorter average insert size (for example, ~1.5 kb for a typical human transcriptome), which severely impairs PacBio CCS throughput. A previous study showed that a total of 11 Sequel SMRT Cells 1M were required to generate 5.2 million reads using ScISOr-Seq for characterizing 6000 single cells^16^. The low yields of existing ScISOr-Seq methods have led to high costs and hindered their wide applications.

**Fig. 1:**
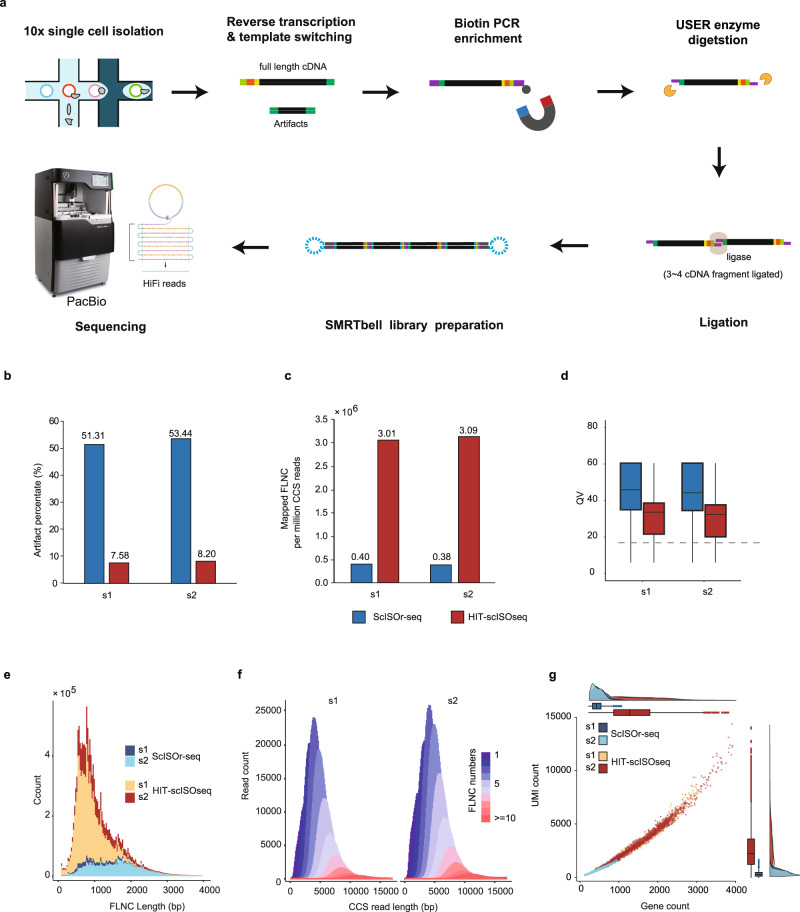
Overview of the workflow and the performance of HIT-scISOseq. **a** Schematic diagram of the experimental steps of HIT-scISOseq, consisting the following steps: (1) Single-cell cDNA library construction; (2) cDNAs amplification via PCR with a biotinylated primer at their 3′ ends; (3) Biotinylated cDNAs enrichment with streptomycin magnetic beads; (4) USER enzyme digestion to produce sticky ends and multi-cDNA fragment ligation; (5) SMRTbell library preparation and sequencing. **b** Comparison of the percentages of artifact reads between ScISOr-Seq (blue) and HIT-scISOseq (red); either method includes two biological replicates (s1 and s2). **c** Comparison on the number of mapped FLNC reads between ScISOr-Seq (blue) and HIT-scISOseq (red). **d** Comparison of the sequence quality between ScISOr-Seq (blue, s1 *n* = 1,582,427, s2 *n* = 1,271,713) and HIT-scISOseq (red, s1 *n* = 2,870,070, s2 *n* = 3,506,141). The center line: median; boxes: first and third quartiles; whiskers: 5th and 95th percentiles. **e**, **f** Comparison of the FLNC lengths (**e**) and number of FLNC per CCS (**f**) between ScISOr-Seq and HIT-scISOseq. **g** Distributions of gene counts (*x*-axis) and UMI counts (*y*-axis) for ScISOr-Seq and HIT-scISOseq. The box plots (s1 ScISOr-Seq *n* = 1658, s2 ScISOr-Seq *n* = 1408, s1 HIT-scISOseq *n* = 1776, s2 HIT-scISOseq *n* = 1599, the center line: median; boxes: first and third quartiles; whiskers: 5th and 95th percentiles.) and density plots are shown on the top and to the right of the scatter graph. Source data are provided as a Source Data file.

To overcome the throughput limitation of ScISOr-Seq, we develop a single-cell isoform sequencing method, HIT-scISOseq (Fig. 1a), for high-throughput and high-accuracy single-cell RNA isoform sequencing. HIT-scISOseq employs two extra steps for that purpose, TSO artifact removal and cDNA concatenation. In the TSO artifact removal step, HIT-scISOseq uses a PCR-based biotin-assisted capture procedure to remove TSO artifacts and enrich cDNA sequences containing poly(A) tails. Lebrigand et al. suggested that a PCR-based biotin-assisted capture procedure could deplete cDNAs lacking poly(A) tail, but they didn’t apply in their study^12^. An alternative to our approach, published while our approach was under review, is the preferential amplification of non-TSO-artifacts^9^. In the second step, we concatenate multiple cDNA amplicons into long SMRTbell inserts to match the PacBio CCS capacity, which significantly increases the base yield. HIT-scISOseq can generate >10 million long-reads with a single Sequel II SMRT Cell 8M, representing up to ten times the yield of the ScISOr-Seq approach. We demonstrate the efficacy of HIT-scISOseq by sequencing the transcriptomes of 3375 corneal limbus cells (Supplementary Table 5) and using the dataset to detect cell-type-specific isoform expression.

## Results

### HIT-scISOseq design

Droplet-based single-cell RNA sequencing, as performed by the 10× Genomics Chromium system, is commonly used as a scalable solution for cDNA library construction in ScISOr-Seq. The 10× Genomics system uses microfluidic partitioning to capture mRNA in single cells, and then combines TSOs and reverse transcription reactions to prepare small-volume cDNA libraries (Supplementary Fig. 1). About 50% of the libraries are composed of barcode-free TSO artifacts (Fig. 1b, Table 1), which will cause a similar proportion of effective CCS read loss. To remove these artifacts, HIT-scISOseq utilizes a biotinylated PCR primer to hybridize with the desired cDNAs; then we capture the biotinylated cDNAs from PCR amplification using streptavidin beads (Fig. 1a). The capture step can significantly reduce the percentage of TSO artifacts (Fig. 1b) from ~50% (ScISOr-Seq) to ~8% (HIT-scISOseq).

**Table 1:** Performance of ScISOr-Seq, Linked-scISOseq and HIT-scISOseq in corneal limbus samples

	Sample	ScISOr-Seq		Linked-scISOseq	HIT-scISOseq	
		s1	s2	s1	s1	s2
Raw Data	Polymerase reads count (M)	4.95	4.30	5.02	4.74	5.69
	Yield of polymerase reads (GB)	499.77	415.52	365.12	383.74	438.64
	Avg. polymerase reads length (Kb)	101.06	96.66	72.78	80.88	77.06
	Yield of subreads (GB)	487.53	405.99	361.45	379.87	434.44
	Avg. subreads length (Kb)	1.55	1.68	3.64	3.46	3.61
CCS Reads	CCS reads count (M)	4.02	3.38	3.70	3.43	4.23
	Yield of CCS reads (GB)	8.04	7.12	16.56	16.75	21.62
	Avg. CCS reads length (Kb)	2.00	2.11	4.48	4.89	5.11
	Avg. CCS reads passes	70	64	21	23	20
	Avg. CCS reads QV	0.97	0.97	0.95	0.95	0.95
FLNC Detection	Linked cDNA count (M)	3.44	2.90	11.57	11.64	14.84
	FLNC count (M)	1.60	1.29	5.25	10.47	13.23
	NFL count (M)	0.07	0.06	0.14	0.28	0.39
	Artifact RNA count (M)	1.76	1.55	6.18	0.88	1.22
	FLNC percentage (%)	46.59	44.53	45.34	89.99	89.15
	NFL percentage (%)	2.10	2.03	1.24	2.43	2.66
	Artifact cDNA percentage (%)	51.31	53.44	53.42	7.58	8.20
FLNC Mapping	Mapped FLNC Count (M)	1.59	1.29	5.20	10.34	13.05
	Mapped FLNC percentage (%)	99.44	99.50	99.05	98.75	98.65
	Avg. FLNC mapping coverage (%)	99.13	99.14	98.88	98.90	98.83
	Avg. FLNC mapping identity (%)	98.45	98.39	97.60	97.74	97.59
	Avg. collapsed FLNC length (Kb)	2.37	2.47	2.18	2.22	2.24

For raw data, the rows show (from top to bottom): (i) total polymerase read count (million) for each sample; (ii) sum of all polymerase read bases (gigabase) for each sample; (iii) average polymerase read length (kilobase) of each sample; (iv) sum of all subread bases (gigabase) in each sample; and (v) average subread length (kilobase) of each sample. For CCS reads, the rows show (from top to bottom): (i) total CCS read count (million) for each sample; (ii) sum of all CCS read bases (gigabase) in each sample; (iii) average CCS read length (kilobase) of each sample; (iv) average CCS read passes in each sample; (v) average CCS read QV (Phred 33) in each sample. For FLNC detection, the rows show (from top to bottom): (i) total linked cDNA (defined as linked cDNA in each CCS read) count (million) in each sample; (ii) total FLNC read count (million) in each sample; (iii) total non-full length (NFL) read count (million) in each sample; (iv) total artifact cDNA count (million) in each sample; (v) percentage of FLNC in linked cDNAs of each sample; (vi) percentage of NFL in linked cDNAs of each sample; and (vii) percentage of artifact cDNAs in linked cDNAs of each sample. For FLNC mapping, the rows show (from top to bottom): (i) total mapped FLNC count (million) of each sample; (ii) percentage of mapped FLNC in total FLNC of each sample; (iii) average mapping coverage of mapping FLNC in total FLNC of each sample; (iv) average mapping identity of mapping FLNC in total FLNC of each sample; and (v) average collapsed FLNC reads (defined as the reads after mapping quality filtering and collapsing of redundancy) length (kilobase) in each sample.

Another significant barrier that limits the CCS reads yield is the short insert size of the ScISOr-Seq cDNA library. Under PacBio Sequel II CCS mode, a single DNA polymerase enzyme affixed to the bottom of a zero-mode waveguide (ZMW) nanoscale well only amplifies a single DNA molecule in a given period called movie time. As a result, the average length of the amplified DNA molecules (library inserts) can determine the final CCS read length and base yield unless it suppasses the threshold of obtaining enough full passes for subreads. PacBio recommends a library insert size of 10–20 kb for the Sequel II CCS mode. Obviously, the short cDNA library insert lengths (~1.5 kb on average for human) of ScISOr-Seq have severely impaired the sequencing capacility of the Pacbio Sequel II system. As a result, the throughput of PacBio ScISOr-Seq is only 20%-30% of that of PacBio genomic DNA sequencing (Table 1). Previous studies have used Gibson Assembly or Golden Gate Assembly to ligate target short or mid-sized DNA fragments (ConcatSeq: ~200 bp, DeCatCounter: ~870 bp) into long SMRTbell libraries for PacBio sequencing^21,22^. However, these methods show a low throughput. There had been no report on ligating the whole-transcriptome cDNA amplicons of uneven lengths for high throughput PacBio sequencing before the preprint release of this study.

To match the capacity of the ZMW in the PacBio Sequel II system, we link multiple cDNAs together to create a long-insert SMRTbell template for downstream Sequel II CCS sequencing. We add a palindromic sequence upstream of both primers for the 10× Genomics cDNA amplification and use the USER enzyme to generate sticky ends at both terminals of cDNAs. Then, multiple cDNAs are joined using DNA ligase (Fig. 1a). After HIT-scISOseq was preprinted^23^, MAS-ISO-seq also used the USER enzyme to create sticky ends for a sequential array structure to be ligated into ~15 kb cDNA concatemers^24^. However, it divides the cDNAs from each sample into 15 tubes for PCR amplification, which increases experimental steps and complexity.

Through taking these steps, HIT-scISOseq leads to dramatic sequencing throughput enhancement compared to the ordinary ScISOr-seq method (Fig. 1c, Table 1). In this study, we also demonstrate that HIT-scISOseq can be used with a droplet-based 10× Genomics Chromium system for single-cell isoform expression analysis.

### Performance of HIT-scISOseq Sequencing Runs

We compared sequencing read outputs among different library preparation methods using the same PacBio Sequel II instrument and SMRT Cell 8M, including ScISOr-Seq (Supplementary Fig. 1), Linked-scISOseq (Supplementary Fig. 2), and HIT-scISOseq (Fig. 1a & Supplementary Fig. 3). Among these methods, ScISOr-Seq is an ordinary library preparation method without TSO artifact removal and cDNA concatenation steps. Linked-scISOseq only includes a cDNA concatenation procedure but no TSO artifact removal step. Comparison of these three methods allowed performance assessment of either optimization step. We evaluated them using the same limbal epithelium cDNA samples, whose transcriptomic profiles had previously been well characterized. We sequenced two samples (s1 and s2) for either ScISOr-Seq or HIT-scISOseq and only one sample (s1) for Linked-scISOseq, adding up to a total of five PacBio Sequel II SMRT Cells. The libraries were prepared following the Iso-Seq sample preparation protocol using the recommended loading concentrations (Supplementary Table 1).

The computational analysis of concatenated cDNAs requires special attention to the physical proximity of multiple cDNAs and the random cDNA strand directions. Therefore, we developed an isoform data analysis pipeline (scISA-Tools, see in Methods) that can confidently identify poly(A) tails, cell barcodes (cellBC), and unique molecular identifiers (UMI), and assign the reads to individual cells and RNA molecules. Based on PacBio’s recommended Iso-Seq data processing procedure, the mapped cDNAs were further classified as full-length non-chimeric (FLNC), non-full-length (NFL), and artifact reads, based on the presence of a poly(A) tail signal and the 5′ and 3′ cDNA primers. Reads with neither the 3′ primer nor the poly(A) tail were referred to as artifact reads.

We conducted the performance assessment for the library construction strategies on four read levels: raw polymerase reads, CCS reads, FLNC reads, and mapped FLNC reads (Table 1). All three methods yielded similar amounts of raw polymerase reads (ranging from 4.30 to 5.69 M), while the percentage of productive ZMWs (P1 percentage metric) ranged from 53.75% to 71.13%. The similar polymerase reads yields among the three methods suggested the high quality of SMRTbell cDNA templates produced by all three methods. Furthermore, the average polymerase read lengths of the three methods were all above 70 kb, suggesting good quality in the instrument runs. Notably, the average lengths of polymerase reads obtained via Linked-scISOseq and HIT-scISOseq were only 70% of those obtained by ScISOr-Seq (Table 1, Supplementary Fig. 7). This may be due to the unrepaired nicks in the linked long-inserts that hamper the polymerase reaction.

All three methods generated a similar abundance of CCS reads, ranging from 3.38 M to 4.23 M and positively correlated to the polymerase read counts (Table 1). Both Linked-scISOseq and HIT-scISOseq generated longer average CCS read lengths (4.48 kb, Linked-scISOseq; 4.89 kb for the s1 sample and 5.11 kb for the s2 sample, HIT-scISOseq), which were more than double those generated by ScISOr-seq (Table 1, Supplementary Fig. 7). The average CCS read lengths were similar to the average insert lengths of the sequencing libraries (Table 1, Supplementary Fig. 4, and Supplementary Fig. 7). Although the library insert sizes for Linked-scISOseq and HIT-scISOseq are longer than those for ScISOr-Seq, both methods achieved an average of over 20 full passes per CCS read (Table 1, Supplementary Fig. 5). While longer insert sizes resulted in fewer average full passes and lower average read accuracies for HIT-scISOseq (Fig. 1d), ≥75% of HIT-scISOseq reads still had QV values ≥20 (Fig. 1d). Supplementary Fig. 6 demonstrates that most multi-FLNC concatemers also maintained high sequencing quality (QV ≥20). This high consensus accuracy allowed us to demultiplex HIT-scISOseq reads based on 10× Genomics cellular barcodes, and it was found that >93% of the HIT-scISOseq FLNC reads could be successfully assigned to individual cells with a CCS QV ≥0.95 (Supplementary Table 4).

Both Linked-scISOseq and HIT-scISOseq generated a larger number of FLNC reads than ScISOr-Seq did (Table 1). Notably, HIT-scISOseq produced a much lower number of artifact cDNA reads (7.58%, sample s1; 8.20%, sample s2) than Linked-scISOseq (53.42%) and ScISOr-Seq (51.31%, sample s1; 53.44%, sample s2). This result indicates that the capture procedure of HIT-scISOseq effectively removes the majority of TSO artifact reads. After removing artifact reads, the three methods had similar FLNC reads ratios [FLNC/(NFL + FLNC)].

We then aligned the FLNC reads against the reference genome for comparison. HIT-scISOseq produced 6.5× (10.34 M for s1) and 10.1× (13.05 M for s2) mapped FLNC reads per SMRT Cell compared to ScISOr-Seq, and up to 2.0× (s1) mapped FLNC reads compared to Linked-scISOseq (Table 1). The TSO artifact removal procedure increased the mapped FLNC reads by ~2.0-fold, and the cDNA concatenation procedure increased the mapped FLNC reads by factors of 3.3 (s1) to 5.1 (s2); together, a combined 8.3-fold mapped FLNC read increase on average. As a result, the number of single-cell genes and UMI detection levels of HIT-scISOseq were markedly higher than those of ScISOr-Seq (Fig. 1g, Supplementary Table 5). Despite the reads yield difference, FLNC reads from the three methods showed similar mapping rates. More than 98% and 99% of the FLNC reads from HIT-scISOseq and ScISOr-Seq were mappable, respectively (Table 1). The average reference alignment coverages (>98%) and the average alignment identities (>97%) of Linked-scISOseq and HIT-scISOseq FLNC reads are comparable to those of ScISOr-Seq (Table 1).

HIT-scISOseq covered similar ranges of FLNC read lengths and transcript (collapsed reads) lengths compared to ScISOr-Seq, but its median FLNC length and median transcript length were shorter than those of ScISOr-Seq (Table 1, Fig. 1e, and Supplementary Fig. 7c). We observed read count increase of HIT-scISOseq compared to ScISOr-Seq throughout all read length intervals (Supplementary Table 3), although the enhancement amplitudes differ among the intervals. HIT-scISOseq produced over 2.6 times (2.61-fold for s1 and 3.64-fold for s2) >1.5 kb FLNC reads than ScISOr-Seq while it had the highest (>10 times) read count enhancement on <1.0 kb reads (Supplementary Table 3), indicating the fragment ligation and PCR amplification steps enriched more short FLNCs. This uneven read count increase had limited impacts on gene expression profiling, since HIT-scISOseq had slightly better consistency with NGS than ScISOr-Seq did (Supplementary Fig. 8). The difference in reads yield between biological replicates may be due to the difference in the percentage of productive ZMW loading and sample quality (Table 1). In addition, by extending the reaction time of the USER enzyme and T4 DNA ligase (Supplementary Table 2), we were able to ligate cDNAs into longer concatemers (Supplementary Fig. 9a, b). Combined with the latest PacBio polymerase binding kit (which is suitable for libraries above 3 kb), HIT-scISOseq was able to obtain up to 30 M FLNC reads per SMRT Cell 8M, including 25.64M with QV ≥20 and 4.93M with QV <20 (Supplementary Table 2).

### HIT-scISOseq assigns cell barcodes with high accuracy

**Fig. 2:**
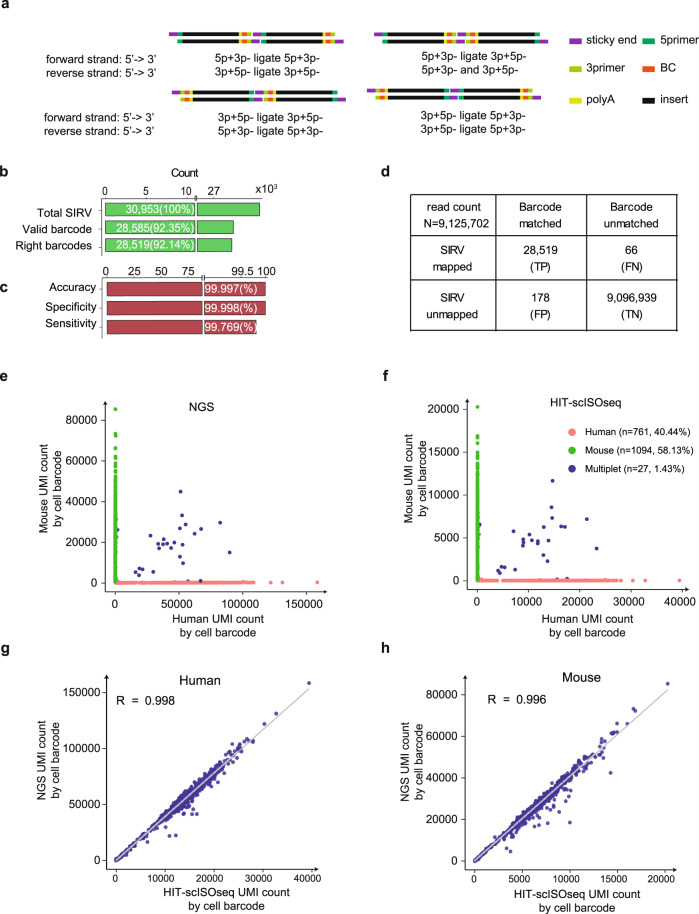
Demultiplexing of HIT-scISOseq concatemers. **a** Four types of terminal combinations present after the ligation of cDNA fragments. These structures are distinguished by different combinations of elements (colored rectangles) as listed in the figure and are defined by the combination of 5′ and 3′ primers used. The 5′ (3′) primer forward and reverse strands are represented by 5p + (3p + ) and 5p- (3p-), respectively. Only fragments with 5′ and 3′ primer sequences at two terminals (5p + 3p- and 3p + 5p-) were regarded as FLNC reads. **b** Barcode assignment statistics for SIRV FLNC reads. The number of all mapped SIRV FLNC reads (Total SIRV) is presented, along with the number of FLNC reads with valid 10× Genomics 16 bp barcodes (Valid barcodes) and that detected with SIRV amplification barcodes (Right barcodes). **c** Accuracy, specificity and sensitivity of barcode assignment. **d** Confusion matrix for barcode assignment. **e, f** Mixed human-mouse test using NGS and HIT-scISOseq. Human and mouse cells were mixed at equal concentrations. Red dots indicate human-specific cells; green dots indicate mouse-specific cells. Only 1.43% (blue dots) are mixed human-mouse cells. **g**, **h** The UMI count correlation between NGS and HIT-scISOseq of human-specific (Pearson’s correlation coefficient *r* = 0.998, *n* = 761, *p* = 0) and mouse-specific cells (Pearson’s correlation coefficient *r* = 0.996, *n* = 1094, *p* = 0). Source data are provided as a Source Data file.

Accurate demultiplexing of HIT-scISOseq concatemers into single-cell long-reads is important for the successful assignment of cell barcodes. The correct segmentation of FLNCs depends on faithfully recognizing all possible element (primers/cellBC/polyA/sticky end) combinations from ligation at FLNC terminals, which are enumerated in Fig. 2a. Accordingly, although HIT-scISOseq has not applied a complicated sequential array structure like MAS-ISO-seq, scISA-Tools can segment its concatemers accurately.

To evaluate the accuracy and specificity of cell-barcode assignment, we amplified the SIRV Set4 synthesized RNA isoforms with “AAGTCCTTCCAGTCTT + 12 N” barcode labeled PCR primers, which was one base edit distance from the most similar 10× whitelist barcode. After double-strand cDNA synthesis, we mixed 0.1 ng of barcoded SIRV cDNA with 99 ng of cDNA from a 10× Genomics human-mouse cell line mixture cDNA for HIT-scISOseq library preparation and sequencing. After demultiplexing HIT-scISOseq concatenated reads, we used mapped FLNC reads from SIRV and human-mouse mixture to calculate the TP, FP, TN, and FN values (Fig. 2d, Supplementary Fig. 9d), which then allowed the calculation of accuracy and specificity for barcode detection. As shown in Fig. 2b, c, scISA-Tools achieved 99.997% and 99.998% barcode assignment accuracy and specificity, respectively. This experiment further confirmed that our demultiplexing and barcode assignment tools were accurate (Fig. 2d–h).

### HIT-scISOseq gene expression clustering of corneal limbus single cells into cell types

**Fig. 3:**
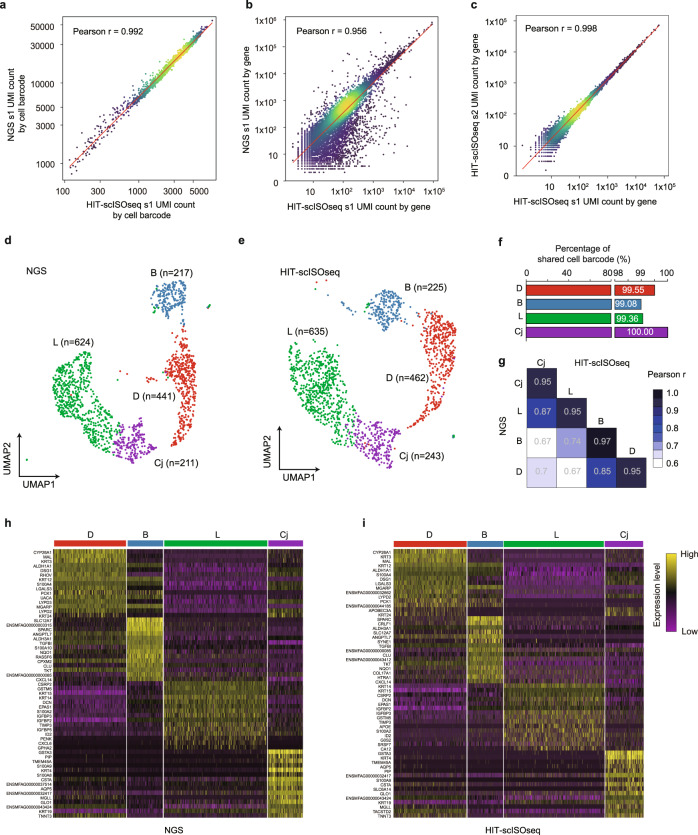
Single-cell gene expression analysis using HIT-scISOseq. **a** Correlation scatter plot showing corresponding UMI counts by cell barcode in NGS (*y*-axis) and HIT-scISOseq (*x*-axis) data (Pearson’s correlation coefficient *r*  = 0.992, *n* = 1676, *p* = 0). The NGS and HIT-scISOseq data sets were generated using cDNA of the same corneal limbus sample s1. Dot colors reflect the local density of data points. **b** Correlation on UMI counts by gene between NGS (*y*-axis) and HIT-scISOseq (*x*-axis) data (Pearson’s correlation coefficient *r* = 0.956, *n* = 14,513, *p* = 0). **c** Correlation of UMI counts by gene between two HIT-scISOseq biological replicate samples (Pearson’s correlation coefficient *r* = 0.998, *n* = 13,663, *p* = 0). **d**, **e** UMAP projection of NGS and HIT-scISOseq data. Gene expression profiles were determined independently for each cell cluster using either NGS or HIT-scISOseq. Both the NGS (**d**) and HIT-scISOseq (**e**) data sets showed that the four main cell populations could be successfully clustered (differentiated cells, denoted by D, are red; corneal basal cells, denoted by B, are blue; limbal stem cells, denoted by L, are green; and conjunctival cells, denoted by Cj, are purple). The undefined cells: which were potentially immune cells (as indicated in Soure Data), and not used in this analysis as since the main focus of this study is on limbal epithelial cells. **f** Bar plot showing the percentages of cell barcodes shared between NGS and HIT-scISOseq data in s1 sample. **g** Heatmap showing the gene expression correlation between NGS and HIT-scISOseq data for each cell cluster in s1 sample. **h**, **i** Heatmaps showing the expression of the top15 marker genes of the four major cell clusters in NGS (**h**) and HIT-scISOseq (**i**) data sets. The color gradient represents log-transformed and row-normalized counts (each row scaled to a maximum of 1). Upper bars represent cell cluster assignments for individual cells. Source data are provided as a Source Data file.

To validate the ability of HIT-scISOseq in distinguishing different cell types, we compared HIT-scISOseq and Illumina short-read RNA sequencing (NGS) on the same single-cell 10× Genomics limbal epithelium cDNA samples, which consisted of several well-defined cell types. There were strong correlations on both the UMI counts by cellBC (Pearson’s *r* = 0.992) and the UMI counts by gene (Pearson’s *r* = 0.956) between the HIT-scISOseq and NGS platforms (Fig. 3a, b and Supplementary Fig. 11a, b). There was also a high concordance (Pearson’s *r* = 0.998) in UMI counts by gene in the HIT-scISOseq data generated from the two biological replicates (Fig. 3c). Moreover, UMAP projection of gene expression data from the two platforms both showed clear boundaries for four distinct cell types (Fig. 3d, e and Supplementary Fig. 11c, d), including conjunctival cells, limbal stem cells, central basal cells, and differentiated cells (Supplementary Table 5–7). The barcoding consistency of the top-ranked 2000 cells between NGS and HIT-scISoSeq was ~99% (Supplementary Fig. 10). The gene expression values obtained for the same cell type showed a high correlation (Pearson’s r >0.95, Fig. 3g & Supplementary Fig. 11f) between NGS and HIT-scISOseq, with the percentage of shared cell barcodes for the same cell type being >99% (Fig. 3f & Supplementary Fig. 11f, Supplementary Table 7). The high concordance of cell barcode counts suggested that HIT-scISOseq can reliably profile the transcriptomes of cells isolated with the 10× Genomics system. We also analyzed the expression of the top 15 marker genes of each cell cluster and discovered similar expression patterns between the two platforms (Fig. 3h, i & Supplementary Fig. 11g, h). These results confirm that the single-cell gene expression profiling results based on HIT-scISOseq are comparable to those by the NGS-based method.

### HIT-scISOseq captures single-cell isoform expression in the corneal limbus

**Fig. 4:**
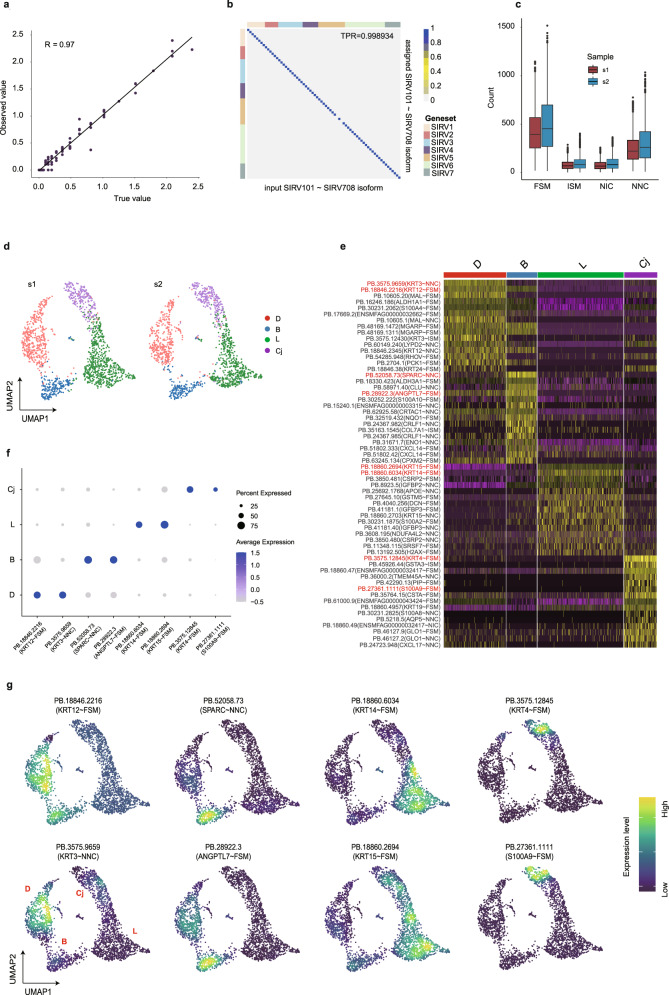
Single-cell isoform-level expression analysis using HIT-scISOseq. **a** Correlation scatter plot between the expected (*x*-axis) and observed abundances (*y*-axis) of the SIRV transcripts (log transformed, Pearson’s correlation coefficient *r* = 0.97, *n* = 62, *p* = 0). **b** Confusion matrix heatmap showing the assignment ratios of SIRV FLNC reads obtained by HIT-scISOseq. The *x*-axis represents the known (true) SIRV isoforms, and the *y*-axis represents the predicted SIRV isoforms from FLNC reads. The ratios of predicted isoform FLNC reads being uniquely assigned to each true SIRV isoform were recorded in the matrix. TPR stands for true positive rate, representing the average value of the diagonal line. **c** The number of FSM (s1 *n* = 1776, s2 *n* = 1599), ISM (s1 *n* = 1776, s2 *n* = 1599), NIC (s1 *n* = 1776, s2 *n* = 1599), and NNC (s1 *n* = 1776, s2 *n* = 1599) isoforms identified and classified by SQANTI3 at the single cell level. The center line: median; boxes: first and third quartiles; whiskers: 5th and 95th percentiles. **d** UMAP of HIT-scISOseq data at the isoform-level. The number of cells in each cluster is the same as that displayed in Fig. 3e. **e** Single-cell marker isoform expression heatmap showing cell-type-specific marker isoform expression among different cell clusters. The color gradient represents log-transformed and row-normalized counts scaled to a maximum of 1. The upper bars denote cell group assignments for individual cells. The eight isoforms marked in red are depicted in **f**, **g**. **f** The Seurat dot plot showing the expression of marker isoforms for each cell type. **g** The Seurat feature-plot showing the single-cell expression of marker isoforms for each cell type. Source data are provided as a Source Data file.

To verify that HIT-scISOseq can accurately quantify isoform expression, we first used SIRV to demonstrate the isoform detection. We performed isoform identification confusion matrix calculations using HIT-scISOseq SIRV isoform data, which showed a confusion rate as low as 0.1066% (1-TPR, Fig. 4b). Next, we evaluated the isoform quantification results by comparing the observed values obtained by HIT-scISOseq with known ERCC isoform abundance data. The abundance measured by HIT-scISOseq was highly consistent with the known composition with a correlation coefficient of 0.97 (Fig. 4a).

We further examed the power of the HIT-scISOseq in identifying and quantifying single-cell isoforms. After quality control and artifact filtering of the corneal limbus data using SQANTI3^25^, we retained four main types of isoforms according to SQANTI3 classification: FSM (full splice match: isoforms that match reference annotations), ISM (incomplete splice match: isoforms whose internal junction sites agree with reference annotations and but 5′ and/or 3′ exons have truncations compared to reference annotations), NIC (novel in catalog: isoforms that have not been annotated but use a combination of known splice sites and exons), and NNC (novel not in catalog: isoforms that contain at least one splice site not annotated). Finally, we retained 29,392 and 31,793 isoforms from the samples s1 and s2, respectively (Supplementary Table 6). Figure 4c showed that at the single-cell level, FSM was the most abundant isoform type in both samples, and there were a considerable number of NNC isoforms, indicating that our data can be used to improve the reference annotation.

Based on isoform-level expression, we observed the same cell clustering patterns as the above gene-level analysis (Fig. 4d). In addition, isoform-level expression was strongly correlated between the two biological replicates (Supplementary Fig. 12). The top 15 marker isoforms of each cell cluster were further analyzed and some of these isoforms were found to be previously unidentified (Fig. 4e). We mapped them to the human reference genome and confirmed that their exon structures are distinct from known isoforms in human annotations (Supplementary Fig. 13). We selected 2 marker isoforms in each cell type for expression pattern verification. Figure 4f, g showed that these marker isoforms did present cell-type-specific expression, supporting that HIT-scISOseq is capable of resolving single-cell isoform expression.

Furthermore, based on our HIT-scISOseq data, we identified differentially expressed isoforms (DEIs) between different cell types in the corneal limbus. Supplementary Fig. 14b demonstrates the expression of four exemplary DEIs belonging to genes *ITM2B*, *DUSP1*, *B2M*, and *HOPX*. The expression of these genes was driven by the major isoforms, but the expression patterns of the DEIs did not match the expression patterns of their corresponding genes and major isoforms (Supplementary Fig. 14b–e). The exon structures of these DEIs also differed from the reference annotation and the major isoforms (Supplementary Fig. 14a), indicating they might have different functions.

Finally, to validate the cell-type-specific isoforms detected by HIT-scISOseq using qPCR, we chose corneal basal cells and conjunctival cells as validation samples. These cells were chosen because they can be obtained from distinct regions of the ocular surface and are representative of different cell types (Supplementary Fig. 15a). We selected four corneal basal cell-specific isoforms and four conjunctival cell-specific isoforms for qPCR validation (Supplementary Table 8), respectively. As shown in Supplementary Fig. 15b–e, the qPCR results showed expression patterns consistent with those obtained from HIT-scISOseq.

## Discussion

This study demonstrates that HIT-scISOseq is a high-throughput, highly accurate method that can be used to characterize isoforms in thousands of single cells. The PacBio Sequel II SMRT Cell 8M has allowed long insert reads (15–20 kb) to be used with high consensus accuracy (>99.9% for HiFi reads). This study shows that the concatenation of multiple cDNAs into a long library can bridge the gap between short libraries and PacBio’s HiFi long-read sequencing capacity. Our experiments demonstrate that HIT-scISOseq is capable of ligating cDNAs into sequences of 15 kb or longer. However, ligation of cDNAs by T4 DNA ligase may generate nicks at the reaction sites. The long-ligated cDNAs are more likely to contain nicks. And the nicks in the sequencing template negatively impact the performance of DNA polymerase in ZWMs. Therefore, in HIT-scISOseq, we employed a PCR step to enrich the nick-free ligation product for sequencing. As a result, the current version of the system enriches ~5 kb concatemers containing 3-4 ligated cDNAs (Fig. 1f). In the future, it will be beneficial to explore methods of reducing DNA nicks, which will enable the construction of high-quality longer insert libraries with more cDNA concatemers, and further improve the throughput of HIT-scISOseq. Moreover, using the BluePippin system to enrich for longer concatenated molecules generated via our method may be an alternative approach for increasing long reads yield.

While this study focused on ligating cDNAs, HIT-scISOseq has used a universal DNA linking protocol, which is possible to target and enrich any sequence of interest, such as LncRNA, circular RNA, 16 S rRNA, and targeted genomic DNA, etc^17,18^. Additionally, the high quality of PacBio CCS reads allows HIT-scISOseq to identify both transcriptional information and somatic mutations at the single-cell level and to reveal more detailed phasing of transcripts at the single-cell level and permit allele-specific expression (ASE) analysis^26^. HIT-scISOseq can also be used in the multiplexed single-cell RNA-sequencing of pooled unrelated individuals, in which natural polymorphisms in long transcripts can be utilized to demultiplex reads and recover sample identity^6^. Furthermore, although the present study only demonstrated that HIT-scISOseq is fully compatible with a commercially available single-cell platform (10× Genomics), it should be readily adaptable to other microwell-based and combinatorial-indexing-based technologies.

## Methods

### Ethical Statement

All animal experiments of this study were conducted by following the ARVO Statement for the Use of Animals in Ophthalmic and Vision Research and received approval from the Ethics Committee of animal experiments at the Zhongshan Ophthalmic Center (Guangzhou, China, acceptance number: 2019-044). The experiments were carried out in two 4-year-old female cynomolgus monkeys (*Macaca fascicularis*) supplied by the Animal Facility of the Zhongshan Ophthalmic Center. The experimental program adheres to ethical standards for animal welfare.

### Monkey limbal sampling experiment

Cynomolgus monkeys (*Macaca fascicularis*) were anesthetized using a mixture of ketamine and xylazine, and topical anesthesia consisted of 0.5% proparacaine hydrochloride (Alcaine; Alcon). Only female monkeys aged 4 years were used. Limbal excision was performed on the right eye, and the left eye was left undamaged. Limbal excision was conducted by lamellar dissection of the limbal zone, 2 mm into the cornea, 2 mm into the conjunctiva, and 100 µm in depth. Biopsy tissues were transferred to cryovials containing Advanced DMEM F-12 and were placed on ice.

### Single-cell dissociation

Dissected limbal tissue was micro-dissected and disaggregated into single cells using Dispase II (Sigma) and collagenase IV (Sigma) at 37 °C under constant rotation. The epithelial layer was isolated from the underlying stroma and was separately digested at 37 °C for 2 h using 2 mL of 1 mg/mL^−1^ collagenase A (Sigma-Aldrich Corp., St. Louis, MO, USA) in Dulbecco’s modified Eagle’s medium (DMEM) containing 10% FBS, 50 μg/mL^−1^ gentamicin, and 1.25 μg/mL^−1^ amphotericin B. The clusters were further digested with 0.25% trypsin and 1 mM EDTA, with gentle pipetting to yield single cells. The cells were filtered through a 30-µm cell strainer and were re-suspended in 60 µL PBS containing 0.04% BSA to obtain a concentration of 1000 cells µl^−1^ for capture on the 10× Genomics Chromium controller.

### 10× Genomics single-cell capture and Illumina library preparation

The dissociated single cells were processed on the GemCode Single Cell Platform per the manufacturer’s recommendations using the Chromium Single Cell 3′ GEM, Library, and Gel Bead Kit v3 (10× Genomics; PN-1000075) with a recovered quantity of ~2000 cells. Illumina library preparation was performed using the Chromium Single Cell 3′ Reagent Kits User Guide (V3 Chemistry). After the cDNA cleanup step (Step 2.1), half of the purified cDNA was used for PacBio library preparation, and the rest was used for downstream Illumina library preparation. Illumina libraries were sequenced on a NextSeq 550 system (SY-415-1002, Illumina) by using NextSeq High Output Kits (150 cycles; 20024907, Illumina) with the following read protocol: read 1, 118 cycles; i7 index read, 8 cycles; read 2, 40 cycles.

### cDNA amplification and capture for PacBio library construction

Eighty nanograms of cDNA products were amplified using five PCR cycles by using KAPA HiFi HotStart Uracil 2 x ReadyMix (Kapa Biosystems) as well as designed PCR primers containing deoxyuracil, one of which was biotinylated.

Forward primer: 5′-ACTAGUAAGCAGTGGTATCAACGCAGAG −3′

Reverse primer: 5′-Biotin-ACTAGUCTACACGACGCTCTTCCGATCT-3′

The PCR products were then purified using 0.8 volumes of Agencourt AMPure XP Beads (Beckman Coulter), quantified using Qubit dsDNA HS Assay Kits (Thermo Fisher), and assessed via Agilent 2100 DNA HS Assays (Supplementary Fig. 4). The barcode-UMI-poly (dT)-flanked cDNAs were captured on streptavidin-coated M-280 Dynabeads using Dynabeads^TM^ kilobaseBINDER^TM^ Kits (60101, Invitrogen, Carlsbad, CA), whereas the unbound cDNAs were removed.

### USER cloning-based ligation of multiple inserts

Complementary DNA products on the Dynabeads were washed with wash buffer and nuclease-free water before being re-suspended in 19 μL reaction buffer containing 2 μL 10× T4 DNA ligase buffer (NEB) and 1 μL USER Enzyme (NEB). The products were then incubated at 37 °C for 20 min, during which the USER enzyme would, cut at the deoxyuracil sites to generate 3′ palindrome overhangs and simultaneously release the cDNA from the M-280 Dynabeads. The reaction tube was placed in a magnetic stand, and the supernatant was transferred to a new tube. One microliter of T4 DNA ligase (NEB, 400,000 U mL^−1^) was added to the reaction mixture, and the resulting mixture was incubated at 16 °C for 10 min to ligate the inserts. The resultant multi-insert library was purified using 0.4 volume of Agencourt AMPure XP Beads (Beckman Coulter) and was then end-repaired and A-tailed using the NEBNext Ultra II End Repair/dA-Tailing Module, with incubation for 15 min at 20 °C and then for 30 min at 65 °C. The cDNA was ligated with 2 μL of a dT-overhang selection adapter (10 μM, annealed with primer 5′-GAACGACATGGCTACGATCCGACTT-3′ and 5′ PHO- AGTCGGATCGTAGCCATGTCGTTC-3′) by using the NEBNext® Ultra™ II Ligation Module (NEB) for 15 min at 20 °C, before being purified with 0.4 volume of Agencourt AMPure XP Beads (Beckman Coulter). Then, 100 ng of the purified products was PCR-amplified for 8-9 cycles using KAPA HiFi HotStart 2x ReadyMix and a selection primer (5′PHO-GAACGACATGGCTACGATCCGACTT-3′) to screen the multi-insert library without ligation nicks. The amplified products were again purified using 0.4 volume of Agencourt AMPure XP Beads (Beckman Coulter) and were assayed using Agilent DNA 12000 Assays. The HIT-scISOseq protocol could be found on https://www.protocols.io/private/7472E845C45C11EC97780A58A9FEAC02.

### PacBio SMRTbell template preparation and sequencing

Amplified PCR products were end-repaired and A-tailed using the NEBNext End Repair/dA-Tailing Module, ligated with a dT-overhang hairpin adapter using the NEBNext® Ultra™ II Ligation Module (NEB), and purified with 0.4 volume of Agencourt AMPure XP Beads (Beckman Coulter) to produce the SMRTbell Template. To remove residual adapters and unligated DNA fragments, 1 μL exonuclease I (NEB), 1 μL exonuclease III (NEB), and NEBuffer 1 (NEB) were added to the library before incubation at 37 °C for 1 h. The products were purified using 0.8 volume of Agencourt AMPure XP beads, eluted with 15 μL elution buffer (10 mM Tris-HCl, pH 8.0), and quantified using Agilent DNA 12000 Kits (Agilent). Sequencing primer annealing and polymerase binding to the PacBio SMRTbell Templates were performed according to the manufacturer’s recommendations (PacBio, US). The library complex was then sequenced using SMRT Cell 8M (PacBio), which was compatible with the Sequel II sequencer.

### Human-Mouse mix sample preparation

Human HEK293T (ATCC, Catalog: CRL-3216; RRID: CVCL_0063) cells and mouse mESC (ATCC, Catalog: CRL-1821; RRID: CVCL_9108) cells were harvested according to the 10× Genomics official protocol (Document CG00054). Afterward, a 1:1 mixture of Human-Mouse cell lines was prepared as per the 10× Genomics official protocol (Document CG00014). The mixture of cell lines was then placed in a −80 °C freezer for at least 4 h, then transfer the cryovials to liquid nitrogen overnight. The next day, after thawing and re-suspending the mixture cell line according to the 10× Genomics official protocol (Document CG00014), the cells were immediately processed using the 10× Genomics Single Cell protocol for targeted capture of 2000 cells.

We prepared cDNA using 1 μL of SIRV-Set 4 (Lexogen) RNA and SuperScript^TM^ II Reverse Transcriptase (Invitrogen) with a barcode labeled oligo dT primer “AAGTCCTTCCAGTCTT + 12 N” as per the manufacturer’s instructions. Following the synthesis of double-stranded SIRV cDNA, 0.1 ng of barcoded SIRV cDNA was added to 99 ng of cDNA from a 10× Genomics human-mouse cell line mixture cDNA to be used as a known cell for HIT-scISOseq library preparation as described above. The cDNA generated by the 10× Genomics system was used to construct HIT-scISOseq libraries as described above.

### RT-qPCR validation

#### RNA isolation and cDNA preparation

Total RNA in each corneal basal cells or conjunctival cells sample was isolated using QIAGEN RNeasy Mini Kit (QIAGEN, Cat# 74104) following the manufacturer’s instructions. Total RNA (50 ng) was used to prepare cDNA using SuperScript^TM^ II Reverse Transcriptase (Invitrogen) with oligo dT according to the manufacturer’s instructions. Briefly, a final volume of 12 µL containing oligo dT primers, dNTP mixture and total RNA was incubated at 65 °C for 5 min and quick chill on ice. Then 5× first-strand buffer, recombinant RNase inhibitor, 0.1 M DTT and SuperScript^TM^ II RT was added to a final volume of 20 µL. Reverse transcription was performed incubating at 42 °C for 50 min followed by inactivation at 70 °C for 15 min. cDNAs were diluted to 60 μL with nuclease-free water.

#### RT-qPCR assay

RT-qPCR was performed in 96-well plates (Axygen) on the StepOnePlus system (Applied Biosystems). Primer sequences and characteristics are shown in Supplementary Table 8. The reaction mix was performed using: 5 μL of TB Green Premix Ex Taq II (Tli RNase H Plus) (Takara, Cat# RR82WR), 1 μL of 5 μM primer mix, 1 μL of diluted cDNA and 3 μL of nuclease-free water. Cycling conditions were 95 °C for 1 min, and 40 cycles of 95 °C for 10 s, 52 °C for 30 s, 68 °C for 30 s. All RT-qPCR experiments were performed using 6 biological and 3 technical replicates.

### HIT-scISOseq data processing pipeline

Since HIT-scISOseq links multiple transcripts together; and multiple cDNA-library-prep-primer sequences can be found in one CCS read, the PacBio official IsoSeq3 pipeline would inherently define HIT-scISOseq reads as “chimeric”; thus, the pipeline was not considered suitable for our analysis. Therefore, a set of analysis tools (https://github.com/shizhuoxing/scISA-Tools) was developed as a pipeline for 10× Genomics ScISOr-Seq read processing. This pipeline included quality control, basic statistics, transcript identification, cell barcode and UMI extraction and correction, isoform clustering, single-cell isoform quantification, and single-cell expression matrix format transformation. This pipeline is not only useful for HIT-scISOseq data but also works well in 10× Genomics systems based on the ScISOr-Seq protocol.

### API for interactive Loupe Browser visualization

Loupe Browser is an established desktop application that allows the interactive visualization of single-cell RNA data from the 10× Genomics platform. scMatrix2CellRangerH5 is a utility developed by this study that can convert a text matrix to an HDF5 format compatible with the CellRanger reanalyze pipelines, which allows “cloupe” files to be generated and visualized in Loupe Browser.

### Single-cell short-read data analysis

For each sample, the 10× Genomics CellRanger pipeline (version 3.1.0) was used to obtain a single-cell expression matrix based on the *Macaca fascicularis* genome and transcriptome (Ensembl Macaca_fascicularis_5.0.99).

### Single-cell isoform sequencing and bioinformatics pipeline

#### Generation of circular consensus sequencing reads

Using SMRT-Link (version 8.0.0.80529), CCS reads were generated with the following modified parameters: “--min-passes 0 --min-length 50 --max-length 21000 --min-rq 0.75”.

#### Generation of single-cell FLNC reads

First, the 5′ and 3′ primers were mapped to CCS reads using NCBI BLAST (version 2.10.0 + )^27,28^ with the following parameters: “-outfmt 7 -word_size 5”. Then, primer BLAST results were used as inputs, and the classify_by_primer utility was employed to extract cell barcodes and UMIs. Finally, FLNC reads were generated with the following parameters: “-min_primerlen 16 -min_seqlen 50”. The functions of the classify_by_primer utility are briefly listed as follows: (1) parsing the 5′ and 3′ primers in CCS reads to obtain FLNC reads, which were then oriented from the 5′ to the 3′ end; (2) trimming 5′ and 3′ primer sequences, trimming the 28 bp sequences followed by the 3′ primers as cell barcodes and UMIs; and (3) trimming the 3′ polyA tail using a sliding window algorithm. As the program was strictly 5′ and 3′ primer paired one after another, each read was oriented. The reads with primers, cell BCs, UMIs, and polyA tails were considered FLNC reads.

#### Genome alignment of FLNC reads

After FLNC detection and trimming procedures were completed, the primers, cell BCs, UMIs, and polyA tails could be identified. The remaining fraction of each FLNC was aligned to the *Macaca fascicularis* genome (Ensembl Macaca_fascicularis_5.0.99) by using minimap2 (version 2.17-r974-dirty)^29^ in spliced alignment mode with the following parameters: “-ax splice -uf --secondary=no -C5”.

#### Cell barcode and UMI correction

A strategy similar to that employed by 10× Genomics CellRanger was adopted. The cellBC correction function in CellRanger was warped as a module in the pipeline, named cellBC_UMI_corrector. This utility could handle long-read data independently, without the need to relate them to short-read information as a guide.

For cellBC correction, CellRanger based on known barcodes for given assay chemistry was stored in a “whitelist” file. The steps are briefly described as follows:

1. The observed frequency of every barcode on the “whitelist” in the data set was counted.
2. For every observed barcode situated 1-Hamming distance (substitution) away from the “whitelist”, the posterior probability that the observed barcode originated from the “whitelist” barcode with a sequencing error at the differing base (based on the base Q score) was computed. Next, the observed barcode was replaced by the “whitelist” barcode with the highest posterior probability exceeding 0.975.

The steps taken for UMI correction are briefly described as follows:

1. Basic quality filtering and correction for UMI sequencing errors with the following restrictions:
  - Must not be a homopolymer, e.g. AAAAAAAAAA;
  - Must not contain N;
  - Must not contain bases with base quality <10.
2. UMIs within 1 Hamming distance (substitution) of a higher-count UMI and sharing a cell barcode in the same gene were corrected to the higher-count UMI.

#### Generation of the single-cell gene count matrix

After mapping FLNCs to the genome, gffcompare (version 0.11.6)^30^ was used and the FLNCs were assigned to Ensemble *Macaca fascicularis* annotation gene models (Ensembl Macaca_fascicularis_5.0.99). The reads were defined as exonic sequences when the class codes equaled ‘“= c k m n j e o”’. This procedure is consistent with the CellRanger pipeline. Next, the scGene_matrix utility was used to generate the single-cell gene expression data for each sample, based on the gffcompare output and corrected cellBC and UMI for each FLNC.

#### Collapsing redundant isoforms

The cDNA_Cupcake (https://github.com/Magdoll/cDNA_Cupcake) Python script “collapse_isoforms_by_sam.py” was used. The “--min-coverage” for minimum alignment coverage and the “--min-identity” for minimum alignment identity default settings were 0.99 and 0.95, respectively. This step ensures the generation of transcripts with high accuracy.

#### Nonredundant isoform classification, coding frame prediction, and UTR detection

SQANTI3 (https://github.com/ConesaLab/SQANTI3)^25^ was used for the characterization, quality control, and rules filter of nonredundant isoforms based on Ensembl *Macaca fascicularis* annotation gene models (Ensembl Macaca_fascicularis_5.0.99). Isoforms were classified as known or novel. SQANTI3 was used to call GeneMarkS-T (version 5.1 March 2014) for nonredundant isoform CDS coding frame prediction and UTR definition.

#### Generation of the single-cell isoform count matrix

After the collapsing procedure, the scIsoform_matrix utility was used to generate single-cell isoform expression quantities in each sample with the following parameters: “-minUMIcount 3”. We further filtered isoforms detected in <5 cells in all samples.

#### Expression matrix quality control

The Seurat R package (version 3.1.5)^31^ was used to perform quality filtering analysis of single-cell genes and isoform expression matrix of each sample. The “min.cells = 5, nFeature_RNA >200, nFeature_RNA <6000, percent.mt <25” command was used for the NGS gene expression matrices of s1 and s2 samples, the “min.cells = 5, nFeature_RNA > 100, nFeature_RNA <3000, percent.mt <25” function was used for the TGS gene expression matrix of s1 samples; and the “min.cells = 5, nFeature_RNA >100, nFeature_RNA <3500, percent.mt <25” command was used for the TGS gene expression matrix of s2 samples.

#### Cell clustering and cell-type annotation

After the quality filtering procedure, the scMatrix2CellRangerH5 utility was used to convert the matrix to the CellRanger h5 format. Then, the CellRanger reanalysis pipeline was used for PCA and cell clustering, with the default parameters. The resulting “cloupe” files were loaded onto the Loupe Browser for adequate manual annotation of cell types and tuning adjustments. After cell-type annotation, the cell type- and cell barcode-associated tables were uploaded into the ‘Seurat’ R package (version 3.1.5) for downstream cell clustering and cell-type marker gene and marker-isoform expression heatmap generation.

#### Differential expression analysis of genes and isoforms

The Seurat R package (version 3.1.5) was used for cell-type gene and isoform marker identification and differential expression analyses.

#### Generation of the isoforms structure view

Selected isoforms of interest were imported as GTF files into IGV (version 2.8.2)^32^ for splicing structure viewing.

### Statistics and reproducibility

This study obtained one sample from the corneal limbus of each of two 4-year-old female cynomolgus monkeys (*Macaca fascicularis*) to create two replicate samples for single-cell RNA sequencing. No statistical method was used to predetermine sample size. No data were excluded from the analyses. The experiments were not randomized. The Investigators were not blinded to allocation during experiments and outcome assessment. All the statistical details for the single-cell RNA sequencing analysis can be found in the figure legends as well as in the Method section.

### Reporting summary

Further information on research design is available in the Nature Portfolio Reporting Summary linked to this article.

## Supplementary information

Supplementary Information

Reporting Summary

## Data Availability

The raw sequencing data generated in this study have been deposited in the Genome Sequence Archive in the BIG Data Center, Beijing Institute of Genomics (BIG, http://gsa.big.ac.cn), Chinese Academy of Sciences, under accession code “PRJCA003458”. The reference genome and gene annotation file (Macaca_fascicularis_5.0.99) were downloaded from Ensembl (https://ftp.ensembl.org/pub/release-99/fasta/macaca_fascicularis/). The remaining data generated in this study are provided in the Supplementary Information. Source data are provided with this paper.

## Source data

Source Data

## Code availability

The HIT-scISOseq analysis pipeline and source code are available from https://github.com/shizhuoxing/scISA-Tools.
