# Supplementary material for: High-throughput and high-accuracy single-cell RNA isoform analysis using PacBio circular consensus sequencing
Source: Nat Commun. 2023 May 6;14:2631. doi: 10.1038/s41467-023-38324-9 (PMC10164132; doi:10.1038/s41467-023-38324-9)
Supplement: Supplementary file 1 — Supplementary Information [file 41467_2023_38324_MOESM1_ESM.pdf]

Supplementary Information

TABLE OF CONTENTS

Supplementary Figures

Supplementary Figure 1. ScISOr-Seq workflow.
Supplementary Figure 2. Linked-scISOseq workflow.
Supplementary Figure 3. HIT-scISOseq workflow.
Supplementary Figure 4. The results of Agilent Bioanalyzer 2100 analysis for each library.
Supplementary Figure 5. The relationship between CCS read accuracy and the number of sequencing passes.
Supplementary Figure 6. The relationship between the number of concatemers and the quality value.
Supplementary Figure 7. Distribution of read lengths for three different types of sequencing data.
Supplementary Figure 8. Comparison of gene expression quantification between HIT-scISOseq, ScISOr-Seq and NGS.
Supplementary Figure 9. Performance of SIRV&Human-Mouse-Mixture data set.
Supplementary Figure 10. Consistency of the top-2000 Cell barcodes between NGS and HIT-scISOseq.
Supplementary Figure 11. Accuracy and reproducibility of HIT-scISOseq gene expression for s2 sample.
Supplementary Figure 12. Reproducibility of the HIT-scISOseq isoform expression.
Supplementary Figure 13. Cross-species verification of marker isoforms.
Supplementary Figure 14. Evaluation of cell-type-specific differentially expressed isoforms using HIT-scISOseq.
Supplementary Figure 15. qPCR validation of cell-type-specific isoforms.

Supplementary Tables

Supplementary Table 1. Quality control reports of ScISOr-Seq, Linked-scISOseq and HIT-scISOseq.
Supplementary Table 2. Quality control reports of two runs of SIRV&HumanMouse HIT-scISOseq.
Supplementary Table 3. FLNC count by length range.
Supplementary Table 4. Cell barcode and UMI correction reports of ScISOr-Seq, Linked-scISOseq and HIT-scISOseq.
Supplementary Table 5. The gene and UMI count in NGS, ScISOr-Seq, Linked-scISOseq and HIT-scISOseq gene matrix.
Supplementary Table 6. The isoform and UMI count in ScISOr-Seq, Linked-scISOseq and HIT-scISOseq isoform matrix.
Supplementary Table 7. Shared cell barcode in each cell type between NGS and HIT-scISOseq.
Supplementary Table 8. qPCR primers for cell-type-specific isoforms validation.

Supplementary Figures

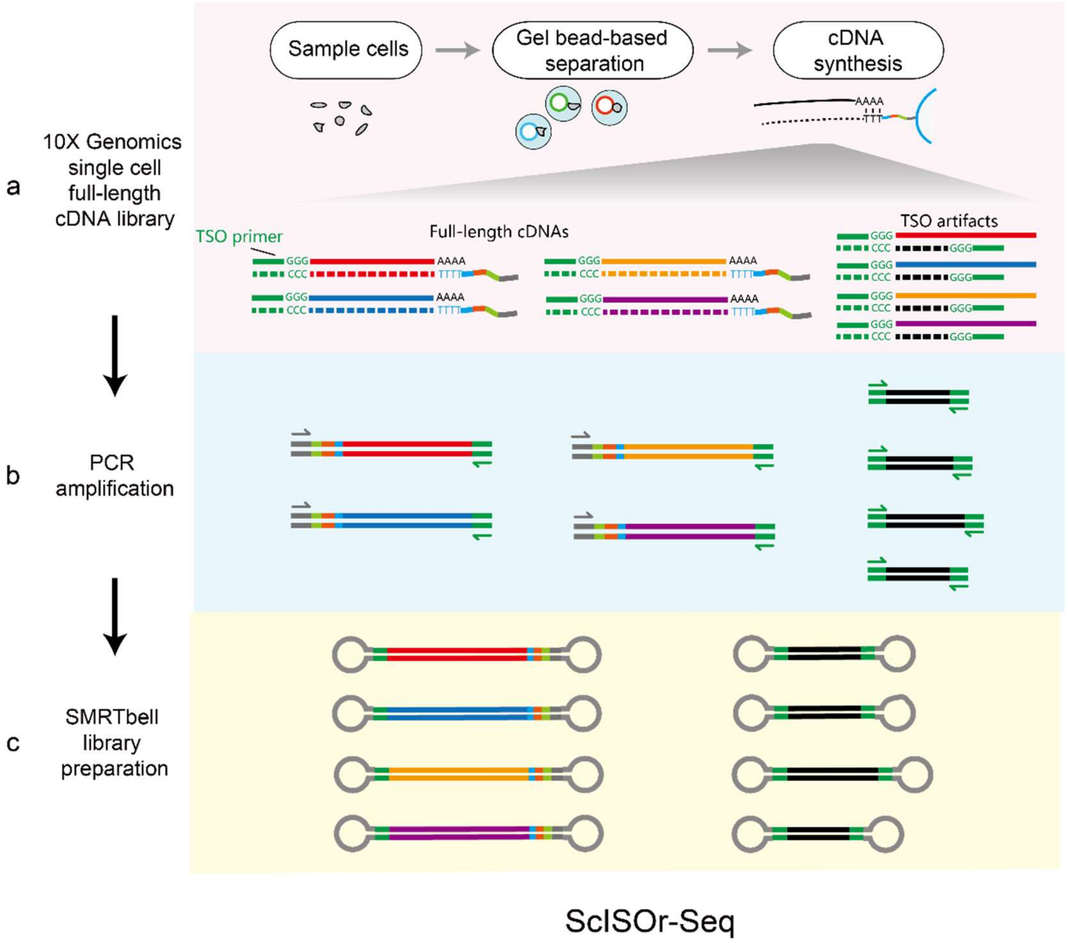

Supplementary Figure 1. **ScISOr-Seq workflow. a** Overview of the 10× Genomics single cell cDNA library construction. **b** cDNA amplification with a 3' PCR primer (gray arrow) and TSO PCR primer (green arrow). **c** SMRTbell library preparation.

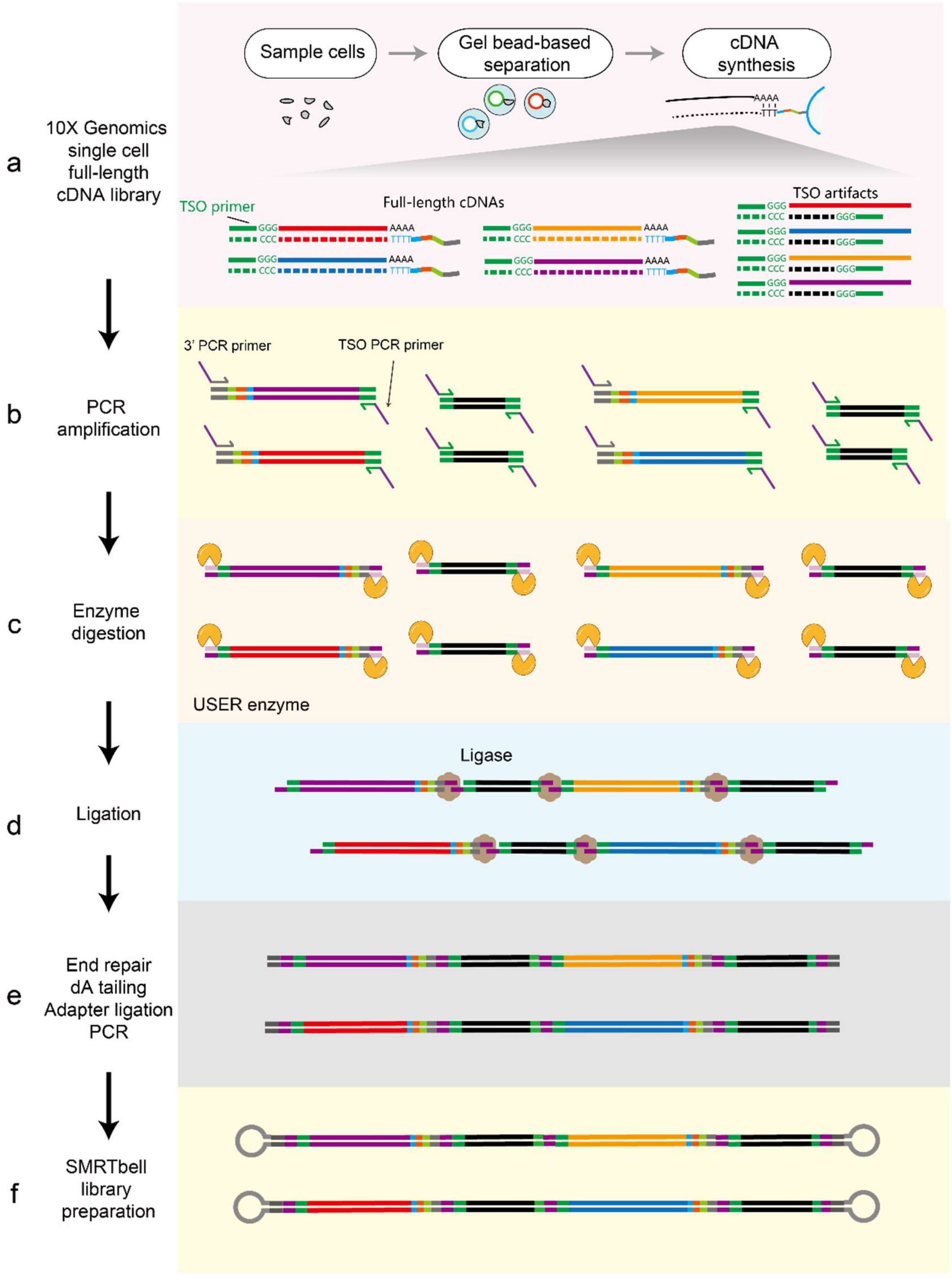

Linked-scISOseq

Supplementary Figure 2. **Linked-scISOseq workflow. a** Overview of 10× Genomics single cell cDNA library construction. **b** cDNA amplification with a 3’ PCR primer and a TSO PCR primer. **c** USER enzyme restriction digestion of the primers at both ends to produce sticky ends. **d** cDNAs ligation. **e-f** End repair, dA tailing, adapter ligation, and PCR enrichment of the ligated cDNAs, followed by SMRTbell library preparation.

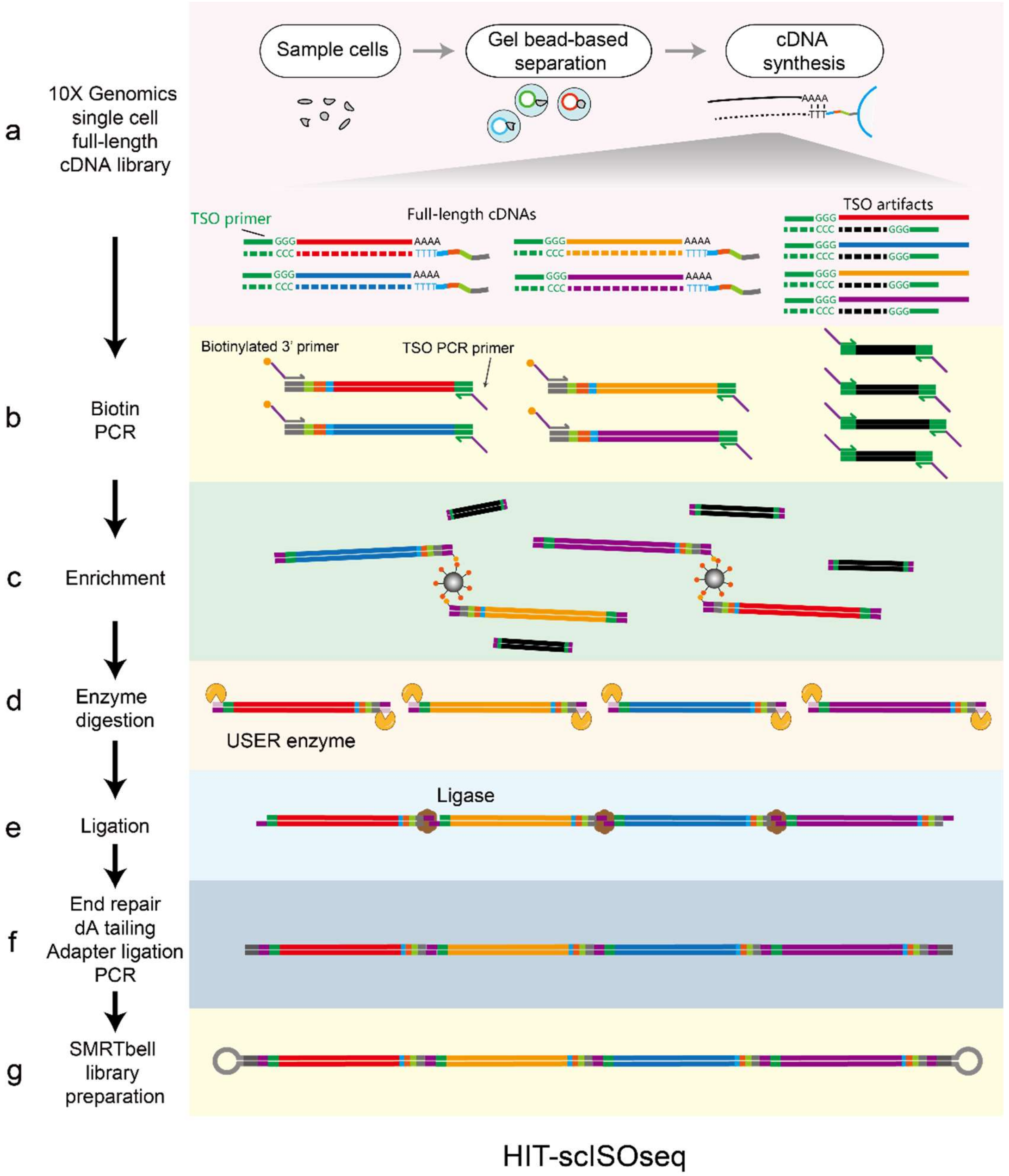

Supplementary Figure 3. **HIT-scISOseq workflow.** **a** Overview of 10× Genomics single-cell cDNA library construction. **b** cDNAs are amplified via PCR with a biotinylated 3’ primer. **c** Biotinylated full-length cDNAs are enriched by streptomycin magnetic beads. **d** Sticky ends are generated by restriction digestion with the USER enzyme. **e** Ligation of cDNAs. **f-g** End repair, dA tailing, adapter ligation and PCR enrichment of the ligated cDNAs, followed by SMRTbell library preparation.

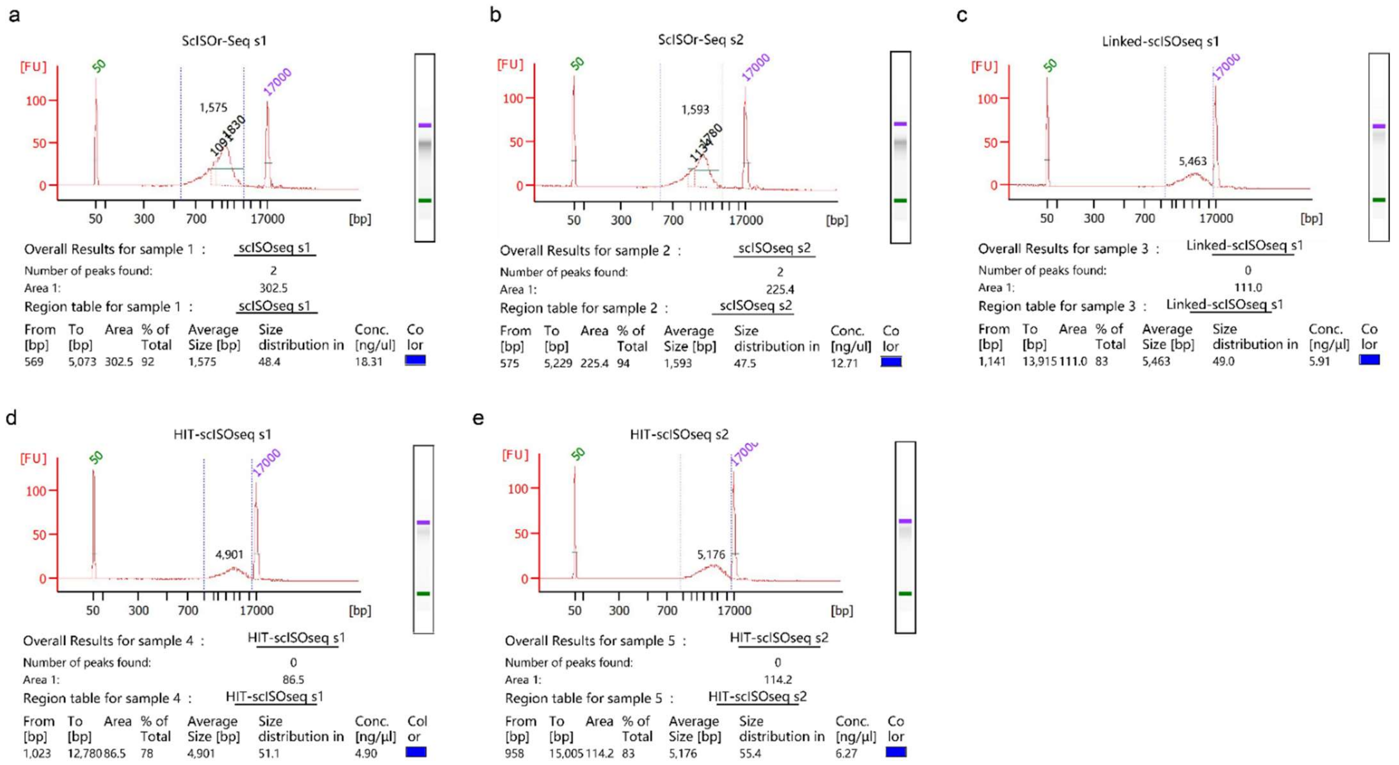

Supplementary Figure 4. The results of Agilent Bioanalyzer 2100 analysis for each libraries. The analysis includes quality control (QC) and quantification results for the ScISOr-Seq (**a-b**) cDNA libraries, Linked-scISOseq (**c**) library, and HIT-scISOseq (**d-e**) libraries.

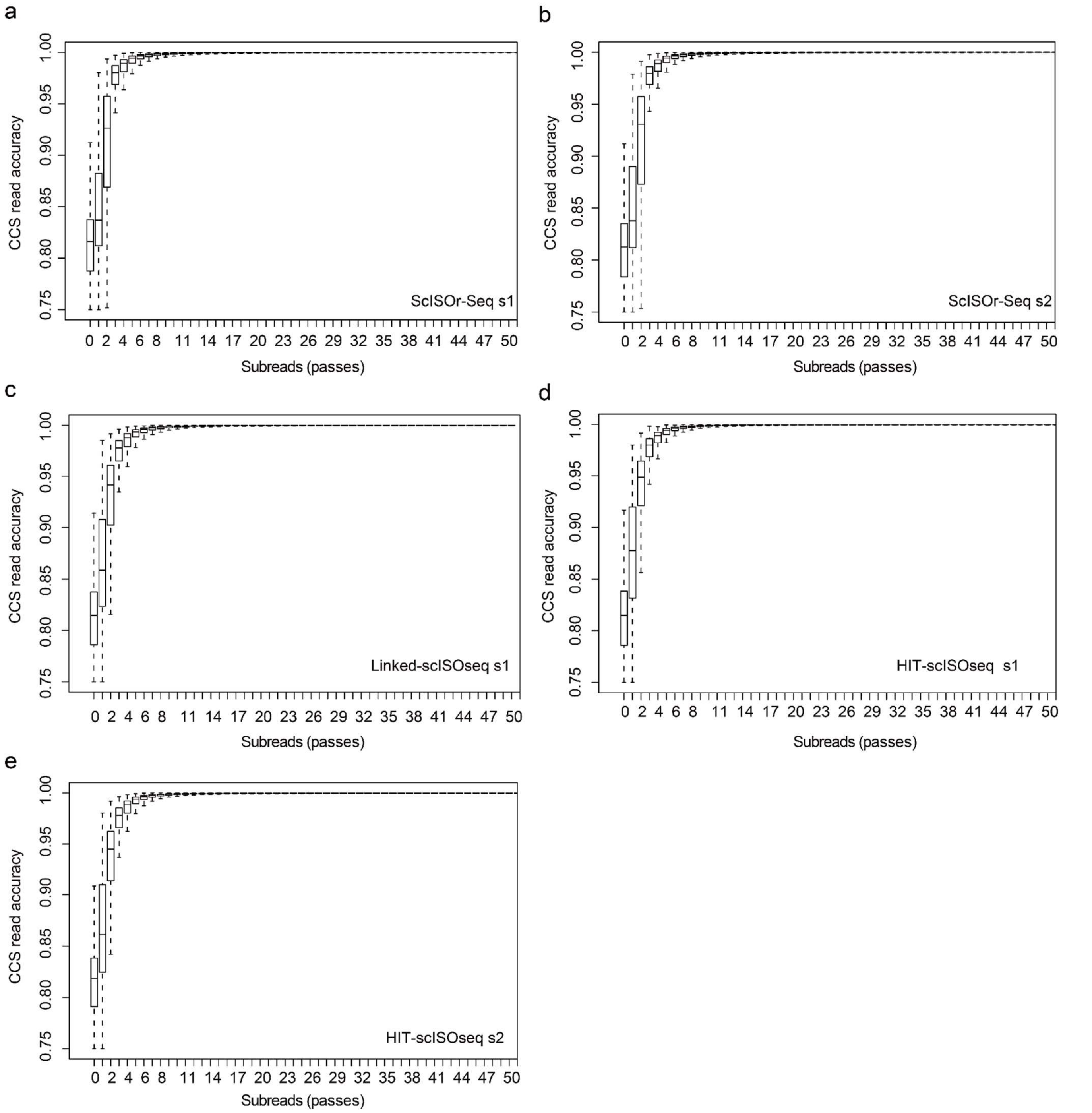

Supplementary Figure 5. The relationship between CCS read accuracy and the number of sequencing passes. The x-axis shows the number of subreads (passes) of SMRTbell, and the y-axis shows CCS consensus read accuracy. **a-b** Samples s1 (*n*=4,016,747) and s2 (*n*=3,380,371) using the ScISOr-Seq method. **c** Sample s1 (*n*=3,697,375) using the Linked-scISOseq method. **d-e** Sample s1 (*n*= 3,427,900) and s2 (*n*=4,226,969) using the HIT-scISOseq method. The center line: median; boxes: first and third quartiles; whiskers: 5th and 95th percentiles..

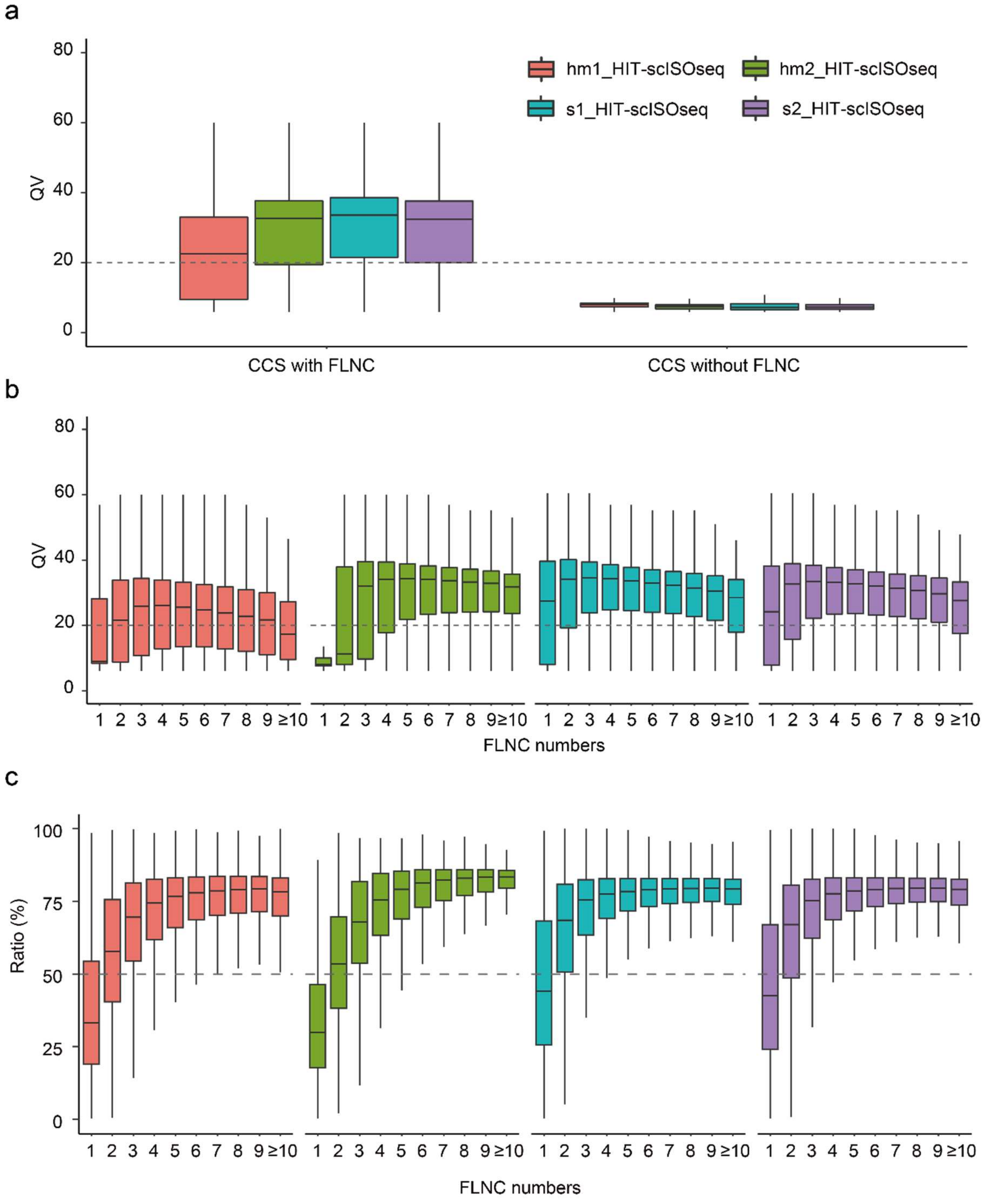

Supplementary Figure 6. **The relationship between the number of concatemers and the quality value.** "hm1" represents SIRV&Human-Mouse sequencing Run1; "hm2" represents SIRV&Human-Mouse sequencing Run2. **a** Distribution of quality values for CCS reads that contain FLNCs and those that do not (hm1 *n*=4,664,865, hm2 *n*=4,864,954, s1 *n*=3,427,900, s2 *n*=4,226,969). **b** Distribution of quality values in relation to the number of FLNCs (hm1 *n*=3,384,305, hm2 *n*=4,442,136, s1 *n*=2,870,070, s2 *n*=3,506,141). **c** Distribution of FLNC length ratio per CCS reads (accumulate of total FLNC length / CCS read length) in relation to the number of FLNCs (hm1 *n*=3,384,305, hm2 *n*=4,442,136, s1 *n*=2,870,070, s2 *n*=3,506,141). The center line: median; boxes: first and third quartiles; whiskers: 5th and 95th percentiles..

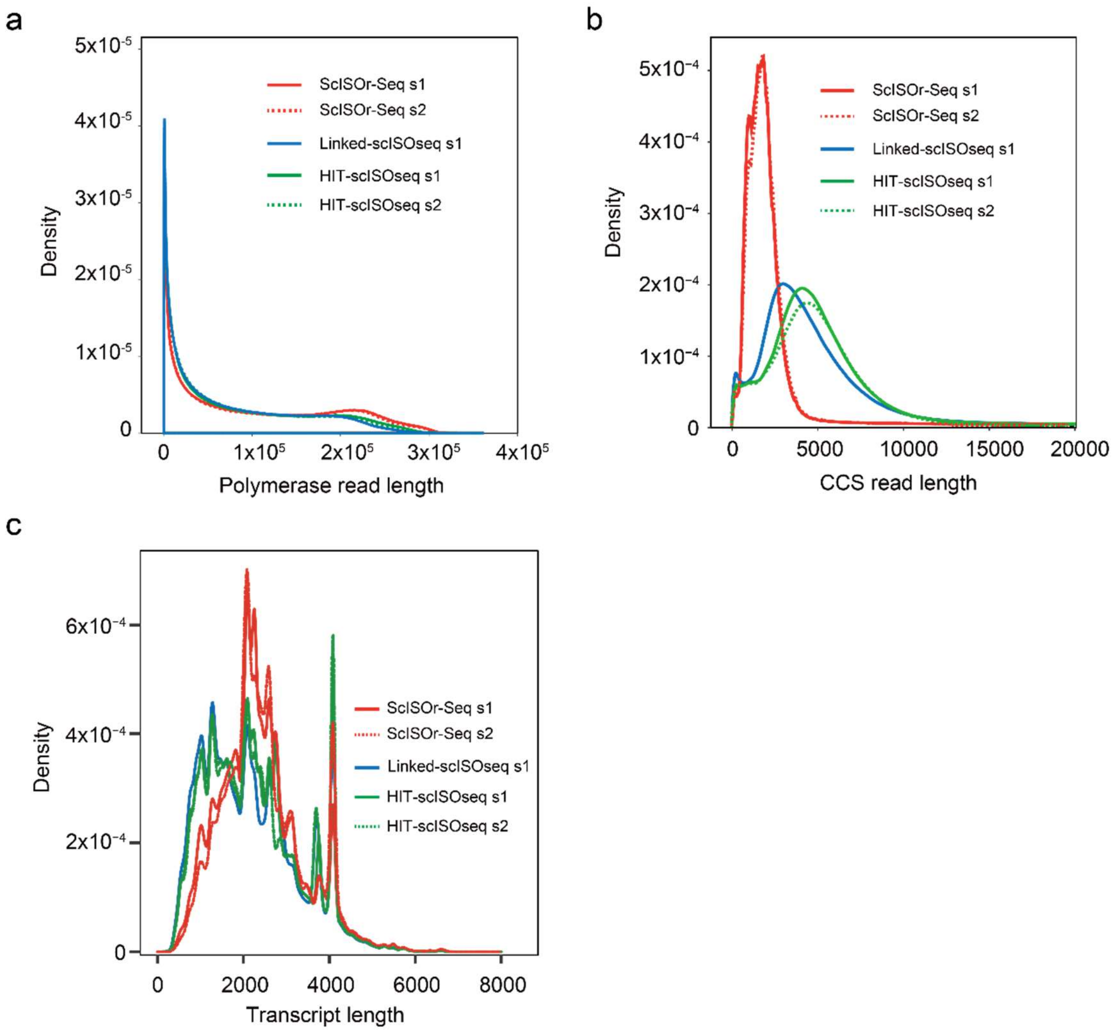

Supplementary Figure 7. Distribution of read lengths for three different types of sequencing data. a Polymerase read lengths, **b** CCS read lengths, and (c) Transcript read lengths.

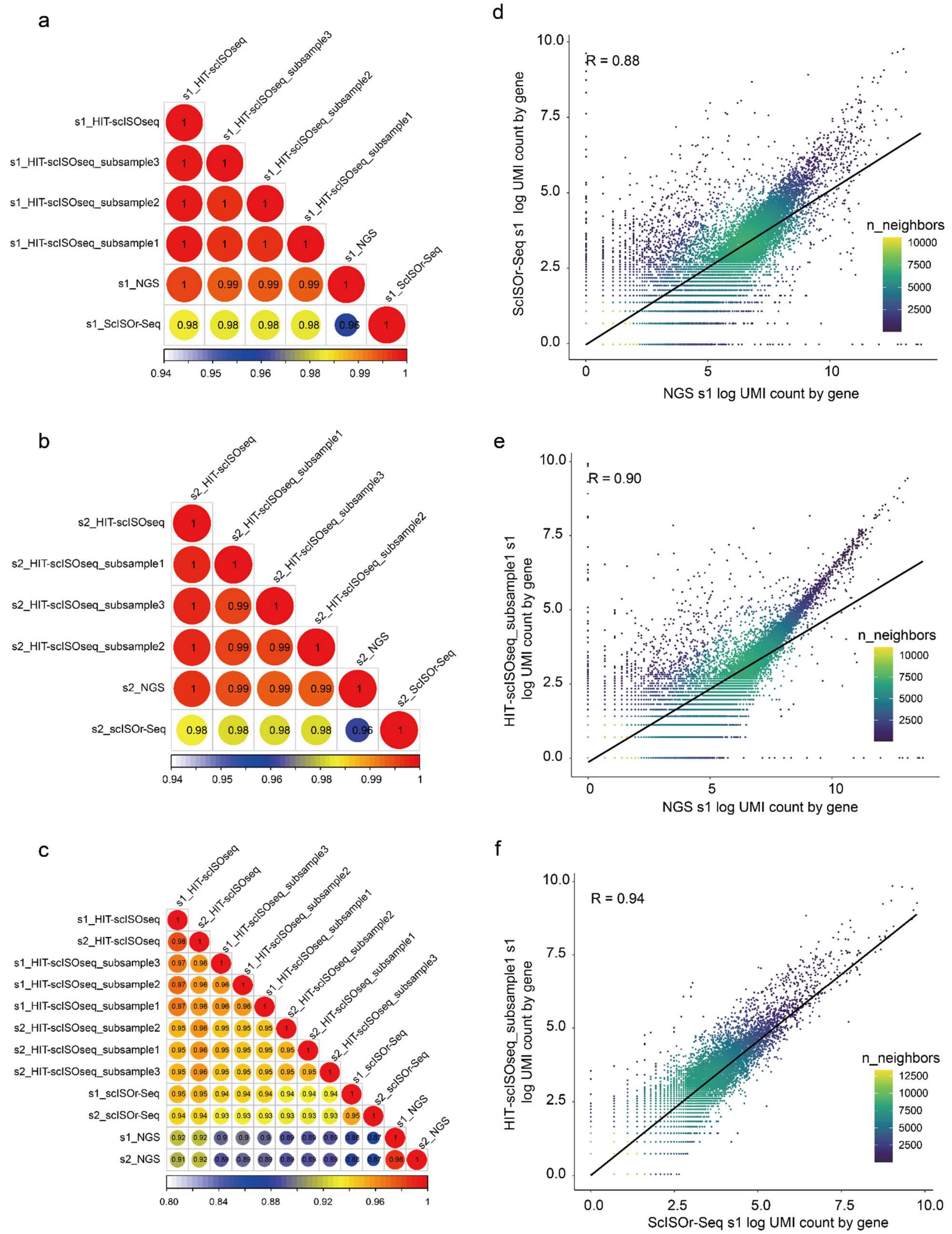

Supplementary Figure 8. Comparison of gene expression quantification between HIT-scISOseq, ScISOr-Seq and NGS. To minimize bias caused by varying cell numbers, we selected the top 2,000 cell barcodes with the highest UMI counts from the NGS data, and down-sampled the HIT-scISOseq data to match the number of ScISOr-Seq FLNCs, with three replicates. **a** Correlation heatmap of log-transformed UMI counts by cell barcode for replicate 1. **b** Correlation heatmap of log-transformed UMI counts by cell barcode for replicate 2. **c** Correlation heatmap of log-transformed UMI counts by gene for replicates 1 and 2. **d-f** Correlation scatter plots of log-transformed UMI counts by gene, comparing NGS with ScISOr-seq (d, Pearson's correlation coefficient r=0.88, n=22,329, p=0), NGS with down-sampled HIT-scISOseq (e, Pearson's correlation coefficient r=0.90, n=22,329, p=0), and ScISOr-seq with down-sampled HIT-scISOseq (f, Pearson's correlation coefficient r=0.94, n=22,329, p=0). Source data are provided in Source Data file.

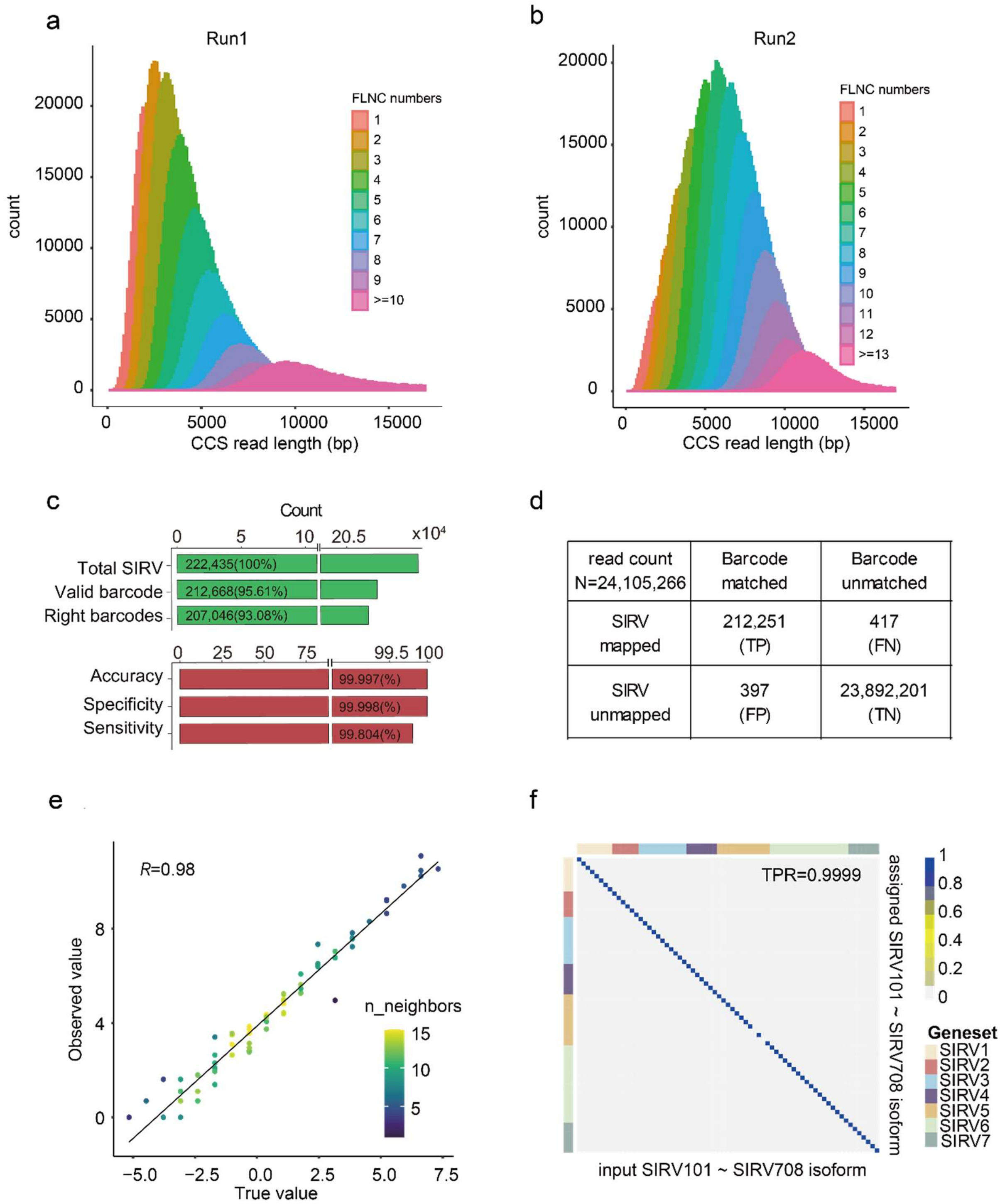

Supplementary Figure 9. **Performance of SIRV&Human-Mouse-Mixture data set.** The Run2 dataset is obtained using the CCS parameter: '--min-passes 0 --min-rq 0.75' (Supplementary Table 2). **a-b** Comparison of the number of FLNC per CCS between two SIRV&Human-Mouse sequencing runs. **c** The number of total split SIRV FLNC, FLNC with validated and accurately split barcodes in SIRV&Human-Mouse sequencing Run2. **d** The accuracy, specificity and sensitivity of barcode assignment in SIRV&Human-Mouse sequencing Run2. **e** Scatter plot shows the correlation between the expected (x axis) and observed abundances (y axis) of the SIRV transcripts in Run2 (Pearson's correlation coefficient r=0.98, n=75, p=0). The values on the plot have been log-transformed. **f** Confusion matrix heatmap showing the assignment ratios of SIRV FLNC reads obtained by SIRV&Human-Mouse HIT-scISOseq sequencing Run2. The x-axis represents the known (true) SIRV isoforms, and the y-axis represents the predicted SIRV isoforms from FLNC reads. The ratios of predicted isoform FLNC reads being uniquely assigned to each true SIRV isoform were recorded in the matrix. TPR stands for true positive rate, representing the average value of the diagonal line. Source data are provided in Source Data file.

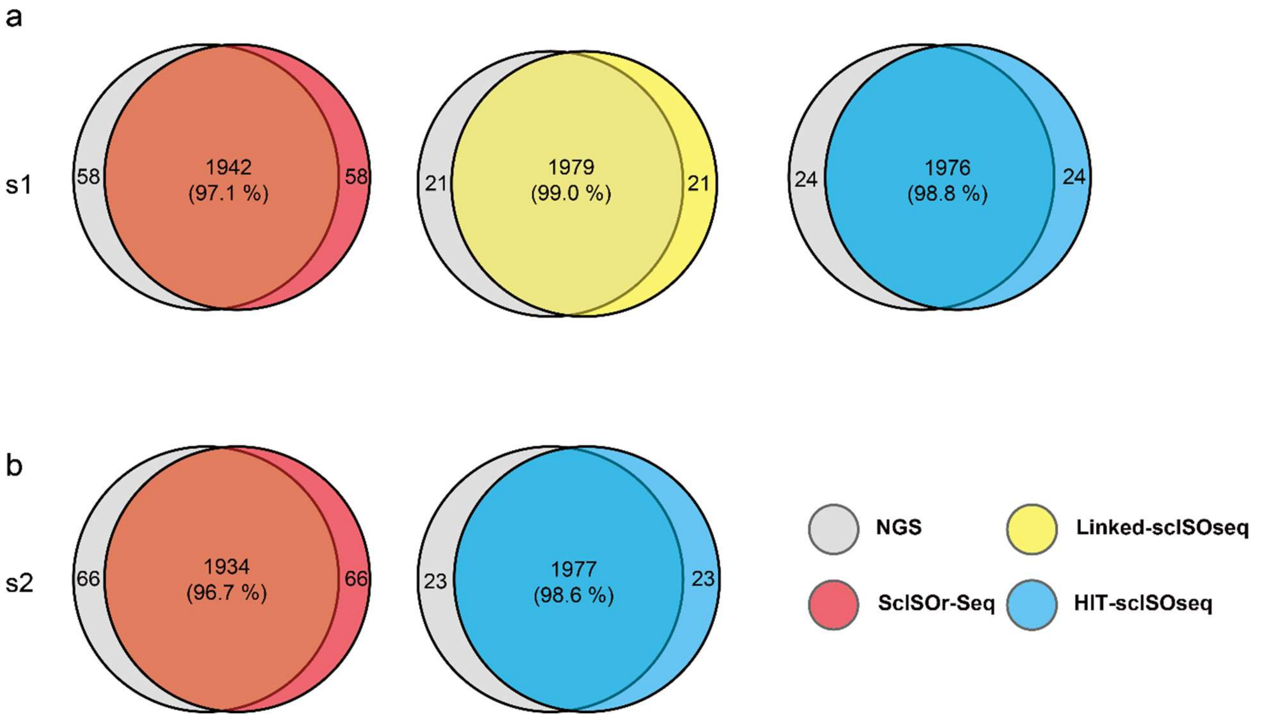

Supplementary Figure 10. **Consistency of the top-2000 Cell barcodes between NGS and HIT-scISOseq.** The numbers (percentages) of shared cell barcodes are shown in the middle areas. NGS method = gray; Linked-scISOseq = yellow; ScISOr-Seq = red; HIT-scISOseq = blue. The upper panel (**a**) shows data from replicate sample s1, and the lower panel (**b**) shows data from replicate sample s2. Source data are provided in Source Data file.

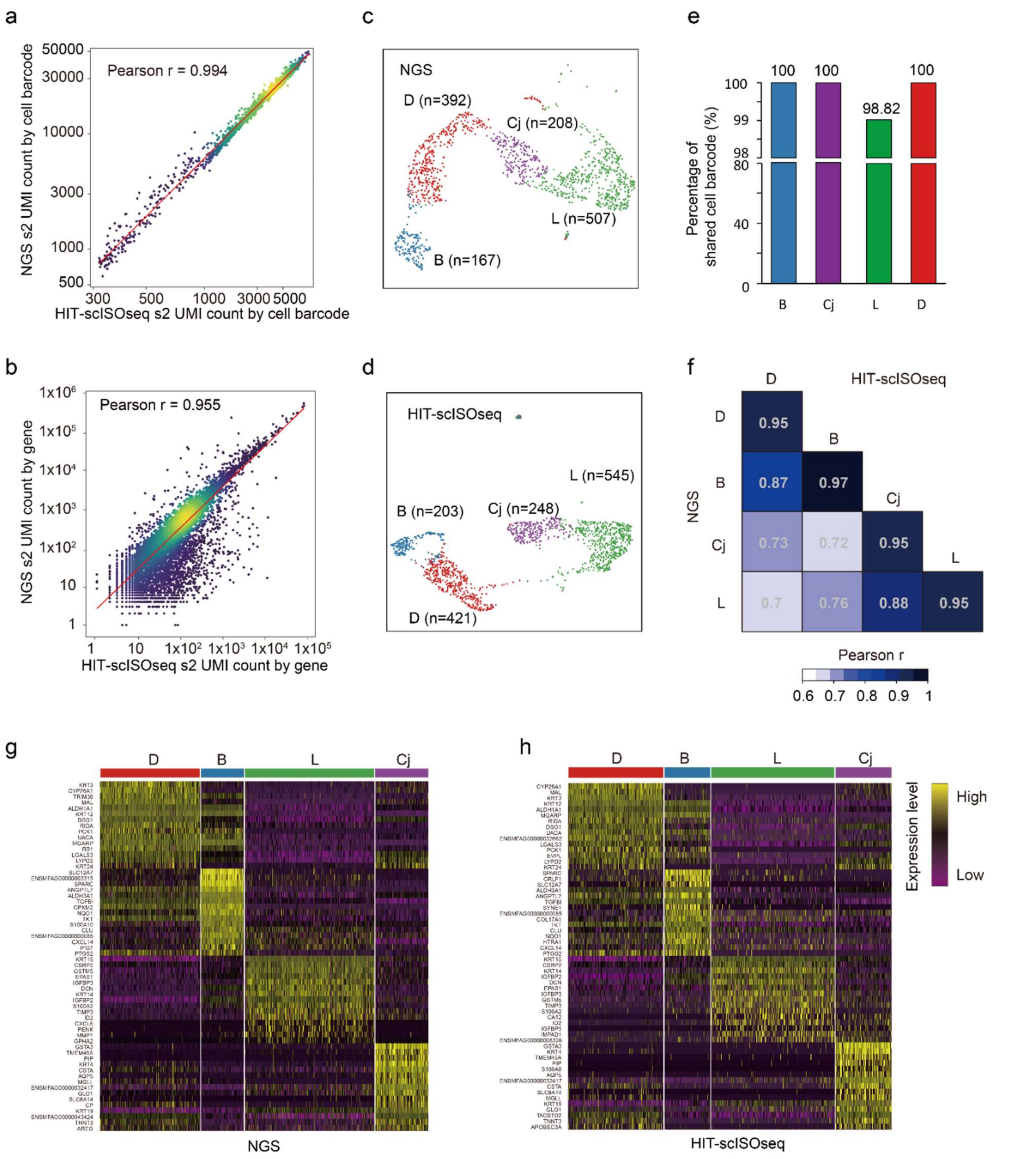

Supplementary Figure 11. **Accuracy and reproducibility of HIT-scISOseq gene expression for s2 sample. a** Correlation scatter plot of NGS (y axis) versus HIT-scISOseq (x axis) UMI counts by cell barcode (Pearson's correlation coefficient *r*=0.994, *n*=1,449, *p*=0). Dot color reflects the local density of data points. **b** Correlation scatter plot of NGS (y-axis) versus HIT-scISOseq (x-axis) UMI counts by gene (Pearson's correlation coefficient *r*=0.955, *n*=14,561, *p*=0). **c-d** UMAP of NGS and HIT-scISOseq data. Gene expression profiles were determined independently for each cell cluster using either NGS or HIT-scISOseq. Both NGS (c) and HIT-scISOseq (d) data sets show that the four main cell populations could be successfully clustered (differentiated cells, indicated by D and red ; corneal basal cells, indicated by B and blue; limbal stem cells, indicated by L and green; and conjunctival cells, indicated by Cj and purple). **e** Bar plot showing the percentage of cell barcodes shared between NGS and HIT-scISOseq data sets. **f** Correlation heatmap showing the gene expression correlation between NGS and HIT-scISOseq data sets for each cell cluster. **g-h** xpression heatmaps of marker genes for the four major cell clusters in the NGS (g) and HIT-scISOseq (h) datasets. The color gradient represents log-transformed and normalized counts scaled to a maximum of 1 per row. Upper bars represent cell cluster assignment for individual cells. Source data are provided as Source Data file.

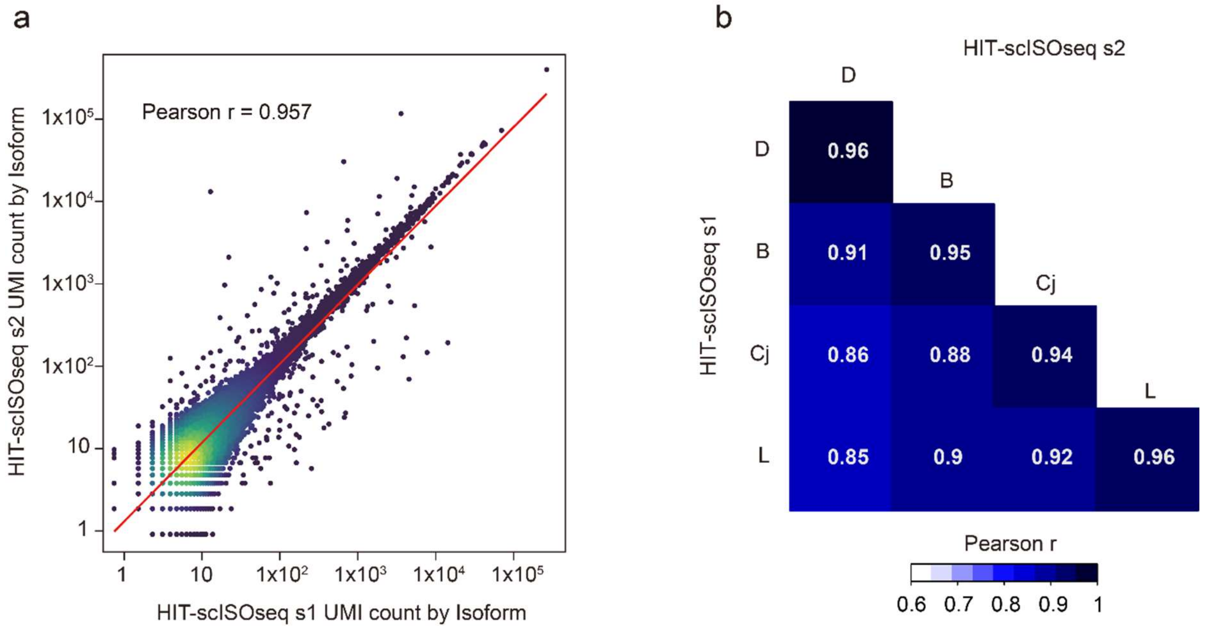

Supplementary Figure 12. **Reproducibility of the HIT-scISOseq isoform expression. a** Correlation scatter plot of UMI counts by isoform between the two replicate samples (*p*=0). Dot color reflects the local density of data points. **b** Isoform expression correlation heatmap of each cell cluster between the two replicate HIT-scISOseq datasets.

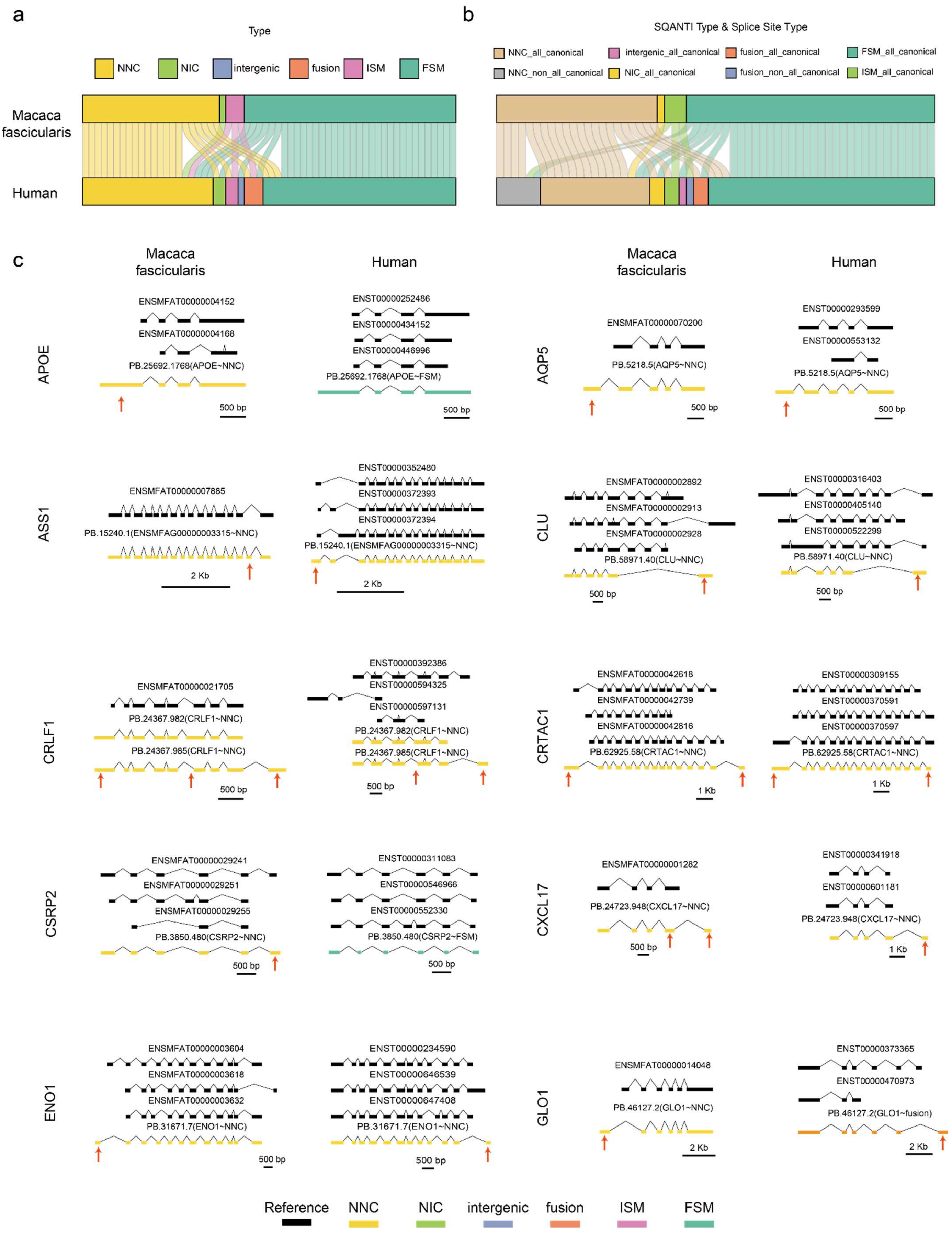
a
Type
NNC
NIC
intergenic
fusion
ISM
FSM
Macaca fascicularis
Human
b
SQANTI Type & Splice Site Type
NNC_all_canonical
intergenic_all_canonical
fusion_all_canonical
FSM_all_canonical
NNC_non_all_canonical
NIC_all_canonical
fusion_non_all_canonical
ISM_all_canonical
c
Macaca fascicularis
Human
Macaca fascicularis
Human
APOE
ENSMFAT00000004152
ENSMFAT00000004168
PB.25692.1768(APOE~NNC)
ENST00000252486
ENST00000434152
ENST00000446996
PB.25692.1768(APOE~FSM)
500 bp
AQP5
ENSMFAT00000070200
PB.5218.5(AQP5~NNC)
ENST00000293599
ENST00000553132
PB.5218.5(AQP5~NNC)
ASS1
ENSMFAT00000007885
PB.15240.1(ENSMFAG00000003315~NNC)
ENST00000352480
ENST00000372393
ENST00000372394
PB.15240.1(ENSMFAG00000003315~NNC)
2 Kb
CLU
ENSMFAT00000002892
ENSMFAT00000002913
ENSMFAT00000002928
PB.58971.40(CLU~NNC)
ENST00000316403
ENST00000405140
ENST00000522299
PB.58971.40(CLU~NNC)
CRLF1
ENSMFAT00000021705
PB.24367.982(CRLF1~NNC)
PB.24367.985(CRLF1~NNC)
ENST00000392386
ENST00000594325
ENST00000597131
PB.24367.982(CRLF1~NNC)
PB.24367.985(CRLF1~NNC)
CRTAC1
ENSMFAT00000042618
ENSMFAT00000042739
ENSMFAT00000042816
PB.62925.58(CRTAC1~NNC)
ENST00000309155
ENST00000370591
ENST00000370597
PB.62925.58(CRTAC1~NNC)
1 Kb
CSRP2
ENSMFAT00000029241
ENSMFAT00000029251
ENSMFAT00000029255
PB.3850.480(CSRP2~NNC)
ENST00000311083
ENST00000546966
ENST00000552330
PB.3850.480(CSRP2~FSM)
CXCL17
ENSMFAT00000001282
PB.24723.948(CXCL17~NNC)
ENST00000341918
ENST00000601181
PB.24723.948(CXCL17~NNC)
ENO1
ENSMFAT00000003604
ENSMFAT00000003618
ENSMFAT00000003632
PB.31671.7(ENO1~NNC)
ENST00000234590
ENST00000646539
ENST00000647408
PB.31671.7(ENO1~NNC)
GLO1
ENSMFAT00000014048
PB.46127.2(GLO1~NNC)
ENST00000373365
ENST00000470973
PB.46127.2(GLO1~fusion)
Reference
NNC
NIC
intergenic
fusion
ISM
FSM

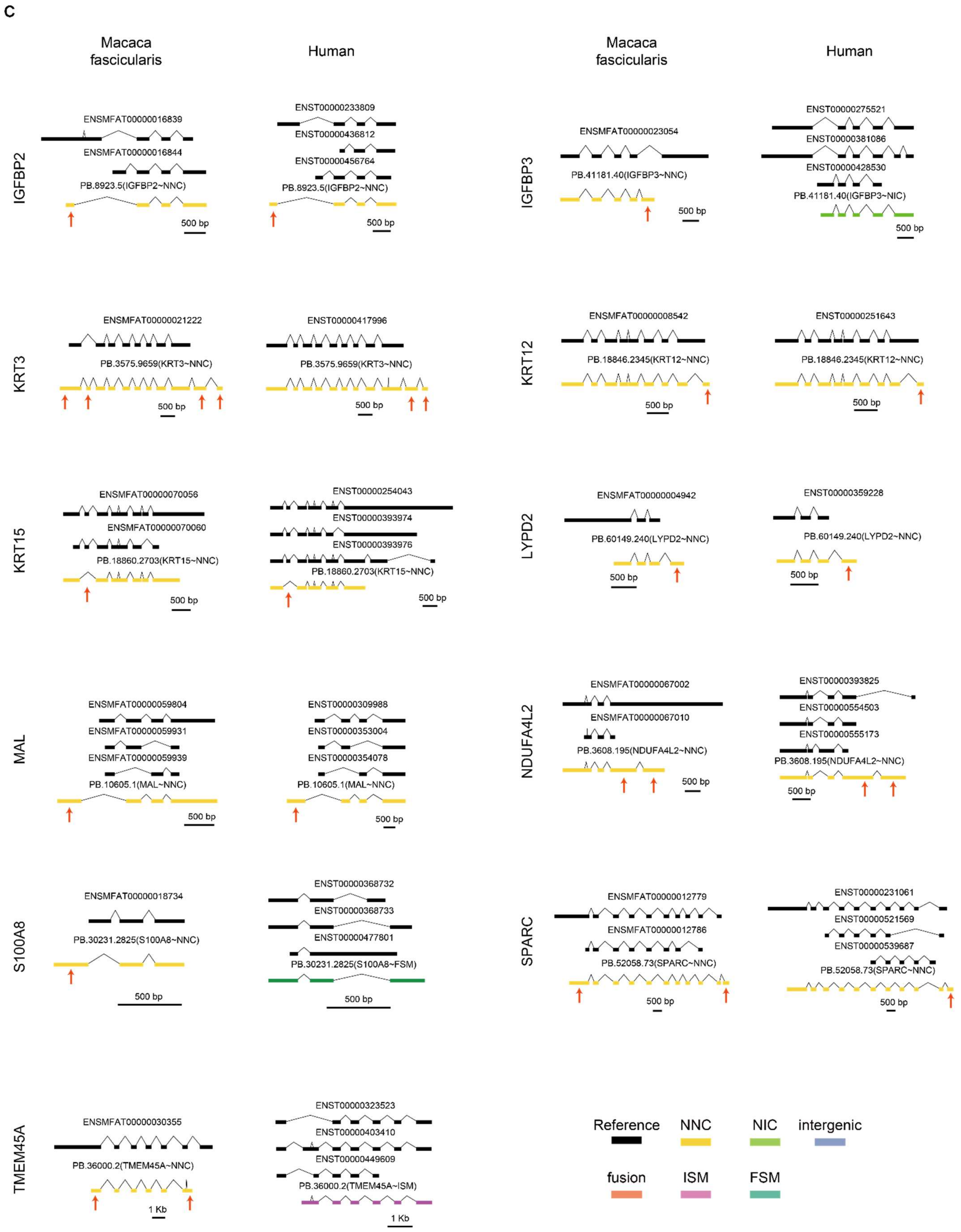

Supplementary Figure 13. **Cross-species verification of marker isoforms. a** Alluvial plot displaying the transitions of SQANTI3 category relationships for marker isoforms between Macaca fascicularis and Humans. **b** Alluvial plot showing the transitions of splice sites between marker isoforms from Macaca fascicularis and Humans. **c** Comparison of the exon structures of NNC marker isoforms with other known isoforms of the corrsponding Humans and Macaca fascicularis genes. The red arrow indicates the novel exon.

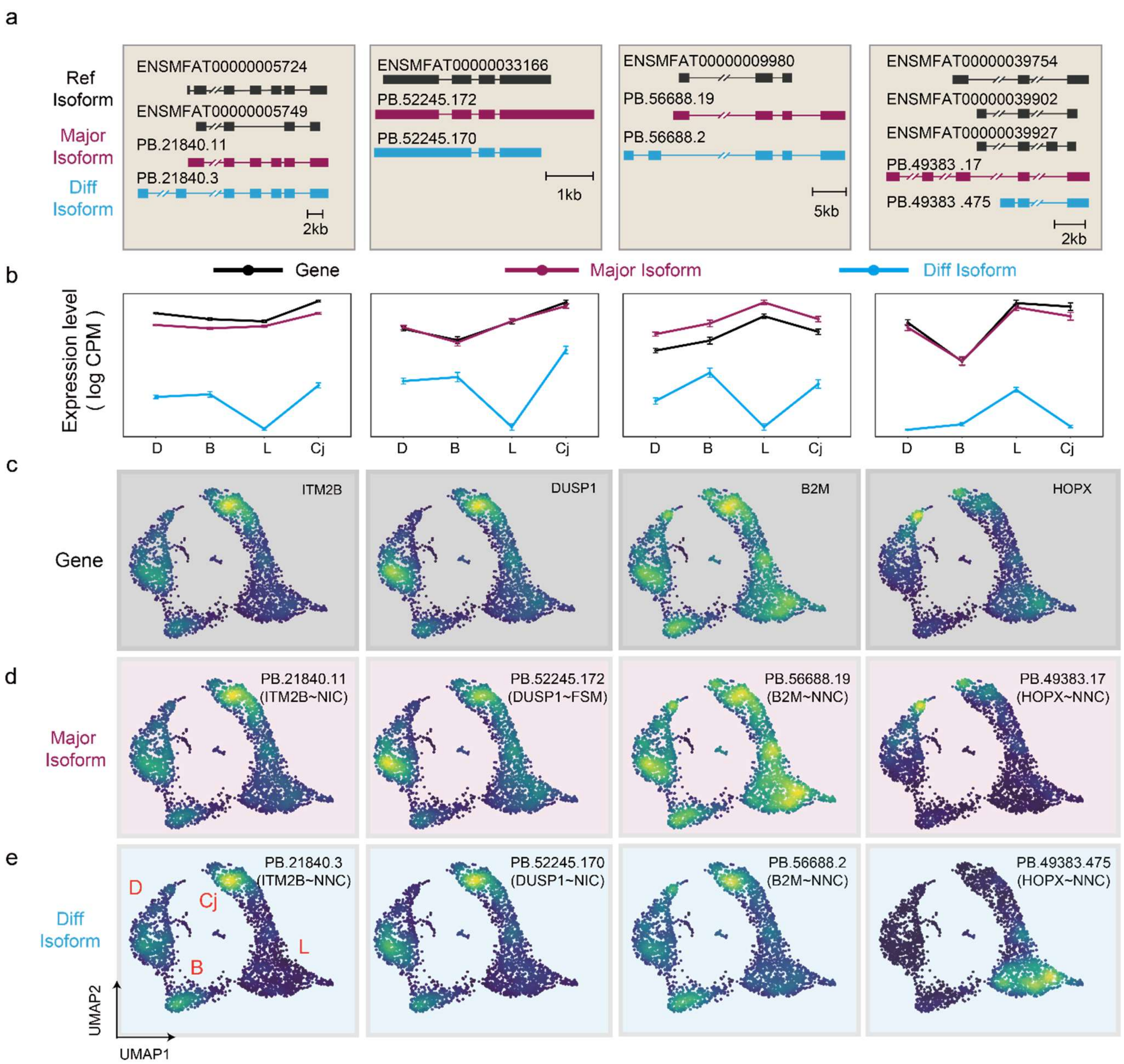

Supplementary Figure 14. Evaluation of cell-type-specific differentially expressed isoforms using HIT-scISOseq. The results of the Seurat FindMarkers analysis for gene expression and isoform expression are being presented in Source Data file. **a** Exon structure of cell-type-specific differential expression isoforms (Diff Isoform, cyan), major isoforms (Major Isoform, purple) and reference isoforms from ensemble annotation (Ref Isoform, black). **b** Expression of genes (black, *n*=2,982), major isoforms (purple, *n*=2,982) and cell-type-specific differential expression isoforms (cyan, *n*=2,982). Data are presented as mean values +/- SEM. **c-e** The feature-plots that illustrate the single-cell expression of genes (c), major isoform (d) and cell-type-specific differentially expressed isoforms (e) for each cell type. It is mentioned in the caption that for comparing with the isoform levels, the UMPA coordinates of the isoforms have been assigned to the gene expression matrix. Source data are provided as Source Data file.

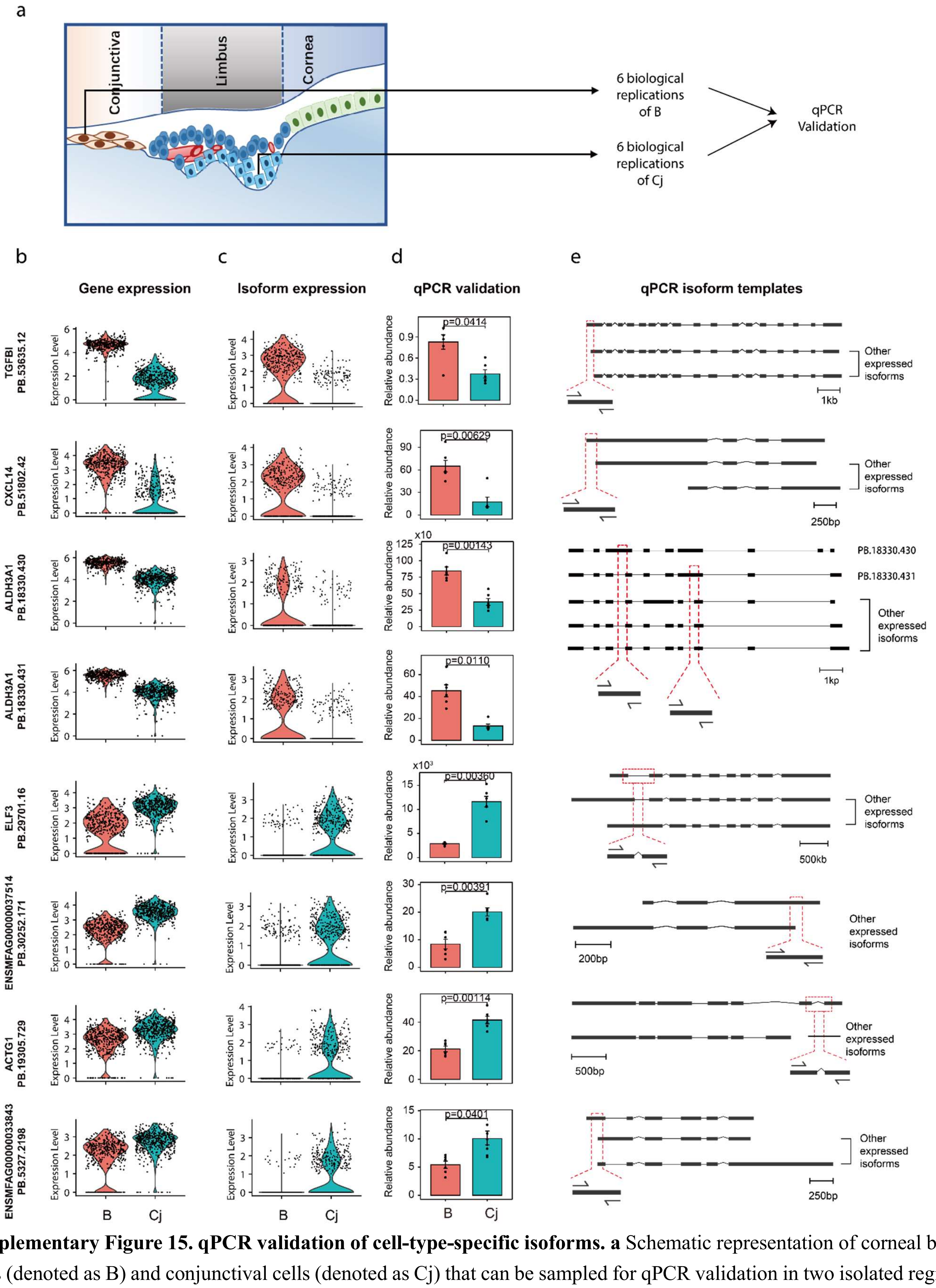

Supplementary Figure 15. qPCR validation of cell-type-specific isoforms. a Schematic representation of corneal basal cells (denoted as B) and conjunctival cells (denoted as Cj) that can be sampled for qPCR validation in two isolated regions of the ocular surface. **b** Single-cell gene expression violin plots of their corresponding genes of B (*n*=428) and Cj (*n*=491). **c** Violin plots of single-cell isoform expression of the 8 selected isoforms of B (*n*=428) and Cj (*n*=491). **d** Bar plot shows the relative abundance (2^-delta (Ct) * 10000) calculated from qPCR results of selected 8 isoforms of B (*n*=6) and Cj (*n*=6). Data are presented as mean values +/- SD. Statistical significance was calculated by two-sided Student's *t*-test, and adjusted *P*-value was calculated using Bonferroni-Holm correction method. **e** Schematic representation of the position of the qPCR template on each isoform is provided and the primer sequence is shown in Supplementary Table 8. Source data are provided as Source Data file.

Supplementary Tables

Sample	ScISOr-Seq		Linked-scISOseq	HIT-scISOseq	
	s1	s2	s1	s1	s2
Sequencing ID	20PW00010	20PW00011	20PW00012	20PW00014	20PW00015
Sequencing Date	2020.03.31	2020.04.07	2020.04.03	2020.04.10	2020.04.09
PacBio Binding Kit	101-789-500 (Kit 2.0)	101-789-500 (Kit 2.0)	101-789-500 (Kit 2.0)	101-789-500 (Kit 2.0)	101-789-500 (Kit 2.0)
PacBio Sequencing Kit	101-826-100	101-826-100	101-826-100	101-826-100	101-826-100
Total Bases (GB)	499.77	415.52	365.12	383.74	438.64
Polymerase reads	4,945,233	4,298,982	5,016,861	4,744,729	5,691,910
Polymerase read N50 (bp)	192,520	189,884	152,456	168,936	159,112
Polymerase mean read length	101,060	96,655	72,778	80,878	77,063
Subreads bases (Gb)	487.53	405.99	361.45	379.87	434.44
Subreads number	314,884,226	242,322,264	99,394,233	109,779,588	120,449,533
Subreads mean read length (bp)	1,548	1,675	3,637	3,460	3,607
Ab 10k (%)	5.74	6.57	12.95	12.62	12.55
Unique molecular yield (GB)	27.83	27.04	44.07	45.42	51.26
P0 (%)	35.73 (2,860,439)	44.23 (3,540,019)	33.72 (2,700,309)	38.75 (3,104,726)	25.54 (2,046,498)
P1 (%)	61.88 (4,953,881)	53.86 (4,310,507)	62.71 (5,022,629)	59.24 (4,746,738)	71.06 (5,693,769)
P2 (%)	2.50 (200,351)	2.05 (164,145)	3.64 (291,733)	2.04 (163,207)	3.42 (274,404)
CPolyReads number	8,607	11,485	5,738	1,989	1,825
CPolyReads mean length (bp)	71,429	53,472	49,963	41,407	49,942
CPolyConcordance	0.85	0.84	0.84	0.84	0.83
Data loss ratio (%)	2.45	2.29	1	1.01	0.96

Supplementary Table 1. Quality control reports of ScISOr-Seq, Linked-scISOseq and HIT-scISOseq. Total Bases (GB): sum of all polymerase reads bases (giga base) in each sample; Polymerase reads: total polymerase reads count of each sample; Polymerase N50 read length: 50% of all polymerase reads are longer than this value; Polymerase mean read length: the mean read length of all polymerase reads; Subreads bases (GB): sum of all subreads bases (giga base) in each sample; Subreads number: total subread count of each sample; Subreads mean read length: the mean read length of all subreads; Ab 10k (%): the percentage of subreads longer than 10kb; Unique molecular yield (GB): sum of all unique molecular reads bases (giga base) in each sample; P0 (%): The percentage of ZMWs that are empty, with no polymerase; P1 (%): The percentage of ZMWs that are productive and sequencing; P2 (%): The percentage of ZMWs that are not P0 (empty) or P1 (productive); CPolyReads number: The number of control polymerase reads; CPolyReads mean length: The mean polymerase read length of control reads; CPolyConcordance: The average concordance (agreement) between the control raw reads and the control reference sequence; Data loss ratio (%): The percentage of loss bases in generating reads from polymerase reads to subreads, i.e., the percentage of base of N or those low quality bases in total bases.

Run Info	Sample	SIRV&HumanMouse Run1	SIRV&HumanMouse Run2		
	Sequencing ID	21P00032	QYT-LXX-PBL212469		
	Sequencing Date	2021.06.05	2021.08.26		
Raw Data	Polymerase reads count	5,563,312	6,004,134		
	Yield of polymerase reads (GB)	192.03	530.70		
	Avg. polymerase reads length (bp)	34,516.41	88,389.16		
	Yield of subreads (GB)	190.57	526.86		
	Avg. subreads length (bp)	4,024.18	6,063.28		
CCS Reads	CCS parameters	'--min-passes 0 --min-rq 0.75'	'--min-passes 0 --min-rq 0.75'	'--all --subread-fallback --min-passes 3 --min-rq 0.99'	
	CCS Type	NA	NA	CCS-Corrected	CCS-Uncorrected
	CCS reads count	4,664,865	4,864,954	3,799,801	2,204,333
	Yield of CCS reads (GB)	21.12	31.19	25.44	41.83
	Avg. CCS reads length (bp)	4,527.70	6,411.59	6,693.82	18,978
	Avg. CCS reads passes	8	15	19	1
	Avg. CCS reads QV	0.92	0.96	1.00	NA
FLNC Detection	Linked cDNA count	15,273,870	29,718,167	26,572,556	6,952,711
	FLNC count	13,506,499	28,148,652	25,641,322	4,929,935
	Mean FLNC per CCS	3.99	6.34	6.77	4.17
	NFL count	487,583	373,777	220,533	457,637
	Artifact RNA count	1,279,788	1,195,738	710,701	1,565,139
	FLNC percentage (%)	88.43	94.72	96.50	70.91
	NFL percentage (%)	3.19	1.26	0.83	6.58
	Artifact RNA percentage (%)	8.38	4.02	2.67	22.51
Experimental Conditions	USER enzyme digestion Time	20min	1h		
	T4 DNA ligase ligation Time	5min	2h		
	PacBio Binding Kit	101-820-500 (Kit 2.1)	101-789-500 (Kit 2.0)		
	PacBio Sequencing Kit	101-826-100	101-826-100		

Supplementary Table 2. Quality control reports of two runs of SIRV&HumanMouse HIT-scISOseq. Polymerase reads count: total polymerase reads count of each sample; Yield of polymerase reads (GB): sum of all polymerase reads bases (giga base) in each sample; Avg. polymerase reads length (bp): the mean read length of all polymerase reads; Yield of subreads (GB): sum of all subreads bases (giga base) in each sample; Avg. subreads length (bp): the mean read length of all subreads; CCS parameters: CCS calling software parameters, '--all --subread-fallback' means: --min-passes 3 --min-rq 0.99 and returen uncorrected CCS Subreads mean read length: the mean read length of all subreads; CCS reads count: total CCS reads count of each sample; Yield of CCS reads (GB): sum of all CCS reads bases (giga base) in each sample; Avg. CCS reads length (bp): the mean read length of all CCS reads; Avg. CCS reads passes: the mean passes of all CCS reads; Avg. CCS reads QV: the mean QV of all CCS reads; Linked cDNA count: total linked cDNAs (which is defined as linked cDNA in each CCS reads) count in each sample; FLNC count: total full-length non-concatemer (FLNC) reads count in each sample; NFL count: total non-full length (NFL) reads count in each sample; Artifact RNA count: total artifact cDNA count in each sample; FLNC percentage (%): percentage of FLNC in linked cDNAs of each sample; NFL percentage (%): percentage of NFL in linked cDNAs of each sample; Artifact RNA percentage (%): the percentage of artifact cDNAs in linked cDNAs of each sample; PacBio Binding Kit: Sequel II binding kit 2.1 (PN: 101-820-500, binding SMRTbell libraries with inserts <3 kb); Sequel II binding kit 2.0 (PN: 101-789-500, binding SMRTbell libraries with inserts >=3 kb).

FLNC Range	ScISOr-Seq				Linked-scISOseq		HIT-scISOseq				Fold-change of FLNC count			
	s1		s2		s1		s1		s2		Linked-scISOseq/ ScISOr-Seq	HIT-scISOseq/ ScISOr-Seq		HIT-scISOseq/ Linked-scISOseq
	Count	Ratio (%)	Count	Ratio (%)	Count	Ratio (%)	Count	Ratio (%)	Count	Ratio (%)	s1	s1	s2	s1
<500bp	87,364	5.45	58,409	4.52	911,495	17.38	1,522,197	14.54	1,873,489	14.16	10.43	17.42	32.08	1.67
500-1,000bp	395,027	24.65	268,800	20.81	2,690,380	51.29	4,893,376	46.73	6,194,722	46.83	6.81	12.39	23.05	1.82
1,001-1,500bp	369,042	23.03	282,219	21.85	964,105	18.38	2,095,301	20.01	2,673,173	20.21	2.61	5.68	9.47	2.17
1,501-2,000bp	409,110	25.53	348,248	26.96	453,127	8.64	1,159,297	11.07	1,477,306	11.17	1.11	2.83	4.24	2.56
2001-2,500bp	220,651	13.77	205,092	15.88	150,635	2.87	446,004	4.26	562,358	4.25	0.68	2.02	2.74	2.96
2,501-3,000bp	84,954	5.30	87,981	6.81	48,707	0.93	172,356	1.65	214,812	1.62	0.57	2.03	2.44	3.54
>=3,001bp	36,262	2.26	40,784	3.16	26,830	0.51	183,925	1.76	231,358	1.75	0.74	5.07	5.67	6.86
All	1,602,410	100.00	1,291,533	100.00	5,245,279	100.00	10,472,456	100.00	13,227,218	100.00	3.27	6.54	10.24	2.00

Supplementary Table 3. FLNC count by length range.

			ScISOr-Seq		Linked-scISOseq	HIT-scISOseq	
		Sample	s1	s2	s1	s2	s1
Cell Barcode (CB)	All	FLNC read count	1,602,410	1,291,533	5,245,279	10,472,456	13,227,218
		CB in whitelist	1,466,251	1,177,044	4,452,458	8,823,984	11,051,565
		CB in whitelist (%)	91.5	91.14	84.89	84.26	83.55
		Corrected CB	13,431	10,814	124,486	248,384	324,201
		Corrected CB (%)	0.84	0.84	2.37	2.37	2.45
		Total number of corrected CB	1,479,682	1,187,858	4,576,944	9,072,368	11,375,766
		Total number of corrected CB (%)	92.34	91.97	87.26	86.63	86
	CB QV>=0.95 (passed)	FLNC read count	1,533,988	1,232,653	4,655,184	9,375,747	11,729,087
		CB in whitelist	1,457,633	1,169,679	4,361,574	8,648,780	10,820,554
		CB in whitelist (%)	95.02	94.89	93.69	92.25	92.25
		CB correction	8,709	6,712	63,986	125,123	159,534
		CB correction (%)	0.57	0.54	1.37	1.33	1.36
		Total number of corrected CB	1,466,342	1,176,391	4,425,560	8,773,903	10,980,088
		Total number of corrected CB (%)	95.59	95.44	95.07	93.58	93.61
UMI	CB passed and UMI passed	FLNC read count	1,533,988	1,232,653	4,655,184	9,375,747	11,729,087
		Discarded UMI	636	397	7,897	19,011	30,719
		Discarded UMI (%)	0.04	0.03	0.17	0.20	0.26
		Uncorrected UMI	1,465,304	1,175,682	4,414,536	8,743,058	10,931,494
		Uncorrected UMI (%)	95.52	95.38	94.83	93.25	93.20
		Corrected UMI	402	312	3,127	11,834	17,875
		Corrected UMI (%)	0.03	0.03	0.07	0.13	0.15
		Total number of passed UMI	1,465,706	1,175,994	4,417,663	8,754,892	10,949,369
		Total number of passed UMI (%)	95.55	95.40	94.90	93.38	93.35

Supplementary Table 4. Cell barcode and UMI correction reports of ScISOr-Seq, Linked-scISOseq and HIT-scISOseq. FLNC read count: number of Full-Length Non-Concatemer reads; For cell barcode correction, "All" means CB have no QV filter, "CB QV>=0.95 (passed)" means have QV filter conditions before CB correction. CB in whitelist: cell barcode can be found directly in the 10x Genomics barcode whitelists; CB in whitelist (%): the percentage of "CB in whitelist" in FLNC read count; Corrected CB: cell barcode have 1- Hamming distance with whitelists; Corrected CB (%): the percentage of "Corrected CB" in FLNC read count; Total number of corrected CB: sum of the "CB in whitelist" and "Corrected CB"; Total number of corrected CB (%): the percentage of "Total number of corrected CB" in FLNC read count; For UMI correction, "CB passed and UMI passed" means have QV filter conditions before UMI correction that CB QV must >=0.95 and have UMI filter conditions after correction that UMI must not "Discarded UMI"; Discarded UMI: means UMI not passed basic quality filtering; Discarded UMI (%): the percentage of "Discarded UMI" in FLNC read count; Uncorrected UMI: means UMI have passed the basic quality filtering but no need correction; Uncorrected UMI (%): the percentage of "Uncorrected UMI" in FLNC read count; Corrected UMI (%): meet the UMI correction conditions and have correction; Corrected UMI (%): the percentage of "Corrected UMI" in FLNC read count; Total number of passed UMI: sum of the "Uncorrected UMI" and "Corrected UMI"; Total number of passed UMI (%): the percentage of "Total number of passed UMI" in FLNC read count.

		NGS		ScISOr-Seq		Linked-scISOseq	HIT-scISOseq	
	Sample	s1	s2	s1	s2	s1	s1	s2
Raw	Number of cells	2,171	2,214	2,171	2,214	2,171	2,171	2,214
	Mean UMI count per cell	14,936	13,740	413	300	1,119	2,138	2,407
	Median UMI count per cell	12,414	10,024	370	232	932	1,795	1,779
	Mean gene count per cell	3,127	2,804	296	221	630	1,042	1,096
	Median gene count per cell	3,189	2,768	280	192	571	976	959
	Total number of genes	16,154	16,088	13,946	13,594	15,161	16,039	16,310
QC filter	Number of cells	1,723	1,525	1,658	1,408	1,735	1,776	1,599
	Mean UMI count per cell	15,924	15,466	514	438	1,313	2,451	3,057
	Median UMI count per cell	14,979	14,683	467	390	1,173	2,208	2,690
	Mean gene count per Cell	3,485	3,333	367	320	745	1,208	1,403
	Median gene count per Cell	3,548	3,525	342	294	691	1,149	1,341
	Total number of genes	13,681	13,588	10,551	9,928	12,039	13,084	13,373

Supplementary Table 5. The gene and UMI count in NGS, ScISOr-Seq, Linked-scISOseq and HIT-scISOseq gene matrix. Number of cell: total number of cell in gene matrix; Mean UMI count per cell: mean UMI detection of each cell in gene matrix; Median UMI count per cell: median UMI detection of each cell in gene matrix; Mean gene count per cell: mean of gene detection of each cell in gene matrix; Median gene count per cell: median of gene detection of each cell in gene matrix; Total number of gene: sum of gene detection of all cell in gene matrix.

		ScISOr-Seq		Linked-scISOseq	HIT-scISOseq	
	Sample	s1	s2	s1	s1	s2
QC filter	Number of cells	1,776	1,599	1,776	1,776	1,599
	Mean UMI count per cell	258.59	213.68	798.34	1,501.36	1,974.14
	Median UMI count per cell	234	191	723	1,349	1,758
	Mean isoform count per cell	183.80	147.00	465.54	813.42	970.02
	Median isoform count per cell	172	133	427.5	753	890
	Total number of isoforms	10,830	8,897	19,711	29,392	31,793

Supplementary Table 6. The isoform and UMI count in ScISOr-Seq, Linked-scISOseq and HIT-scISOseq isoform matrix. Number of cell: total number of cell in isoform matrix; Mean UMI count per cell: mean UMI detection of each cell in isoform matrix; Median UMI count per cell: median UMI detection of each cell in isoform matrix; Mean isoform count per cell: mean type of isoform detection of each cell in isoform matrix; Median isoform count per cell: median type of isoform detection of each cell in isoform matrix; Total number of isoforms: sum of isoform type detection of all cell in isoform matrix.

Sample	Cell type	NGS cell count	HIT-scISOseq cell count	Shared cell barcode count	Percentage of shared cell barcode (%)
s1	D	441	462	439	99.55
	B	217	225	215	99.08
	L	624	635	620	99.36
	Cj	211	243	211	100
s2	D	392	421	392	100
	B	167	203	167	100
	L	507	545	501	98.82
	Cj	208	248	208	100

Supplementary Table 7. Shared cell barcode in each cell type between NGS and HIT-scISOseq. Shared cell barcode means that identical of cells between NGS and HIT-scISOseq data sets are within the same cell type and with the same cell barcode. The percentage of shared cell barcode was calculated as the shared cell barcode count / the NGS cell count.

Isoform ID	Strand	Primer Sequence
PB.53635.12	Forward primer	CTAAGGGGCCCATACCCTTG
	Reverse primer	CCACCCTGTTCTGCACTGT
PB.51802.42	Forward primer	CCAAAATTCTAGACTGCTGCC
	Reverse primer	TACCAGTTTTCTTTCCCAGCA
PB.18330.430	Forward primer	TCGCTACATAGGTGCGTGAC
	Reverse primer	CCAACCCGAGGTCATGTCTG
PB.18330.431	Forward primer	CCGCACCTGATGATGGAGAG
	Reverse primer	GCCTGGATGGTAGAACAGCA
PB.29701.16	Forward primer	TAGAGCCAGGTAGGGGAACG
	Reverse primer	ATGAGGCTACCGGAGTGGT
PB.30252.171	Forward primer	TTAAAACATGAACTGGAGAGTTGG
	Reverse primer	ACACCCAAGATGCATTTTTCTTATT
PB.19305.729	Forward primer	TCACATGGCAGGCACAAGG
	Reverse primer	TGTGCAGCAGGAACCAAGGA
PB.5327.2198	Forward primer	TGCATAAACCCAGGTCACGG
	Reverse primer	AGTAATGGCTCCGACTCTCG

Supplementary Table 8. qPCR primers for cell-type-specific isoforms validation.
