# Supplementary material for: High-throughput and high-accuracy single-cell RNA isoform analysis using PacBio circular consensus sequencing
Source: Nat Commun. 2023 May 6;14:2631. doi: 10.1038/s41467-023-38324-9 (PMC10164132; doi:10.1038/s41467-023-38324-9)
Supplement: Supplementary file 2 — Reporting Summary [file 41467_2023_38324_MOESM2_ESM.pdf]

nature portfolio

Corresponding author(s): Xiao-Chen Bo, Feng Luo, Chong Tang, Chuan-Le Xiao, and Yi-Zhi Liu

Last updated by author(s): Mar 30, 2023

Reporting Summary

Nature Portfolio wishes to improve the reproducibility of the work that we publish. This form provides structure for consistency and transparency in reporting. For further information on Nature Portfolio policies, see our Editorial Policies and the Editorial Policy Checklist.

Statistics

For all statistical analyses, confirm that the following items are present in the figure legend, table legend, main text, or Methods section.

n/a	Confirmed	
☐	☒	The exact sample size (*n*) for each experimental group/condition, given as a discrete number and unit of measurement
☐	☒	A statement on whether measurements were taken from distinct samples or whether the same sample was measured repeatedly
☐	☒	The statistical test(s) used AND whether they are one- or two-sided<br>*Only common tests should be described solely by name; describe more complex techniques in the Methods section.*
☒	☐	A description of all covariates tested
☒	☐	A description of any assumptions or corrections, such as tests of normality and adjustment for multiple comparisons
☐	☒	A full description of the statistical parameters including central tendency (e.g. means) or other basic estimates (e.g. regression coefficient) AND variation (e.g. standard deviation) or associated estimates of uncertainty (e.g. confidence intervals)
☐	☒	For null hypothesis testing, the test statistic (e.g. *F*, *t*, *r*) with confidence intervals, effect sizes, degrees of freedom and *P* value noted<br>*Give P values as exact values whenever suitable.*
☒	☐	For Bayesian analysis, information on the choice of priors and Markov chain Monte Carlo settings
☐	☒	For hierarchical and complex designs, identification of the appropriate level for tests and full reporting of outcomes
☐	☒	Estimates of effect sizes (e.g. Cohen's *d*, Pearson's *r*), indicating how they were calculated

Our web collection on statistics for biologists contains articles on many of the points above.

Software and code

Policy information about availability of computer code

Data collection	No software was used.
Data analysis	This study utilized open software and packages, which are listed below: R (version 3.6.1); Python (version 3.7.6); 10× Genomics Cell Ranger pipeline (version 3.1.0); SMRT-Link (version 8.0.0.80529); NCBI BLAST (version 2.10.0+); minimap2 (version 2.17-r974-dirty); gffcompare (version 0.11.6); cDNA_Cupcake (version 12.5.0); SQANTI3 (version 1.1); Seurat (version 3.1.5); IGV (version 2.8.2); ggplot2 (version 3.3.2). And custom code and scripts generated within this project are available at https://githu b.com/shizhuoxing/scISA-Tools. Sliding window algorithm is programmed by ours in-house perl script, this program is included in https://github.com/shizhuoxing/scISA-Tools/blob/master/bin/classify_by_primer.pl.

For manuscripts utilizing custom algorithms or software that are central to the research but not yet described in published literature, software must be made available to editors and reviewers. We strongly encourage code deposition in a community repository (e.g. GitHub). See the Nature Portfolio guidelines for submitting code & software for further information.

Data

Policy information about availability of data

All manuscripts must include a data availability statement. This statement should provide the following information, where applicable:

- Accession codes, unique identifiers, or web links for publicly available datasets
- A description of any restrictions on data availability
- For clinical datasets or third party data, please ensure that the statement adheres to our policy

The HIT-sclSOseq pipeline analysis tools and source code are available from https://github.com/shizhuoxing/sclSA-Tools. All data sets used in this study were deposited in the Genome Sequence Archive in the BIG Data Center, Beijing Institute of Genomics (BIG, http://gsa.big.ac.cn) with Project Accession No PRJCA003458 [https://ngdc.cncb.ac.cn/bioproject/browse/PRJCA003458]. The reference genome and gene annotation file (Macaca_fascicularis_5.0.99) were downloaded from Ensembl [https://ftp.ensembl.org/pub/release-99/fasta/macaca_fascicularis/].

Human research participants

Policy information about studies involving human research participants and Sex and Gender in Research.

Reporting on sex and gender	Sex and gender were not relevant to this study.
Population characteristics	Population chracteristics was not relevant to this study
Recruitment	Recruitment was not relevant to this study.
Ethics oversight	Ethics oversight was not relevant to this study

Note that full information on the approval of the study protocol must also be provided in the manuscript.

Field-specific reporting

Please select the one below that is the best fit for your research. If you are not sure, read the appropriate sections before making your selection.

☒ Life sciences ☐ Behavioural & social sciences ☐ Ecological, evolutionary & environmental sciences

For a reference copy of the document with all sections, see nature.com/documents/nr-reporting-summary-flat.pdf

Life sciences study design

All studies must disclose on these points even when the disclosure is negative.

Sample size	This study obtained one sample from the corneal limbus of each of two four-year-old female cynomolgus monkeys (Macaca fascicularis) to create two replicate samples for single-cell RNA sequencing. No statistical method was used to predetermine sample size.
Data exclusions	No data were excluded from the analyses.
Replication	Attempts at replication were successful. Two biological replicates were performed in this study. Reproducibility was assessed based on expression profiles and co-clustering of single-cell transcriptome data from different samples.
Randomization	The experiments were not randomized.
Blinding	The Investigators were not blinded to allocation during experiments and outcome assessment. The sequencing experiments and analyses were carried on two biological replicate singleton samples.

Reporting for specific materials, systems and methods

We require information from authors about some types of materials, experimental systems and methods used in many studies. Here, indicate whether each material, system or method listed is relevant to your study. If you are not sure if a list item applies to your research, read the appropriate section before selecting a response.

Materials & experimental systems

n/a	Involved in the study
☒	☐ Antibodies
☐	☒ Eukaryotic cell lines
☒	☐ Palaeontology and archaeology
☐	☒ Animals and other organisms
☒	☐ Clinical data
☒	☐ Dual use research of concern

Methods

n/a	Involved in the study
☒	☐ ChIP-seq
☒	☐ Flow cytometry
☒	☐ MRI-based neuroimaging

Eukaryotic cell lines

Policy information about cell lines and Sex and Gender in Research

Cell line source(s)	Human HEK293T cells (ATCC, Catalog: CRL-3216; RRID: CVCL_0063) cells; Mouse mESC cells (ATCC, Catalog: CRL-1821; RRID: CVCL_9108) cells.
Authentication	All the cell lines were authenticated with short tandem repeat (STR) profiling method.
Mycoplasma contamination	All cell lines tested negative for mycoplasma contamination.
Commonly misidentified lines (See ICLAC register)	No commonly misidentified cell lines were used in the study.

Animals and other research organisms

Policy information about studies involving animals; ARRIVE guidelines recommended for reporting animal research, and Sex and Gender in Research

Laboratory animals	The monkey experiment involved two four-year-old female cynomolgus monkeys (Macaca fascicularis).
Wild animals	This study did not involve wild animals
Reporting on sex	This study was to compare the performance of different methods and the sex of the animals do not affect the results, therefore the sex-based analyses was not considered.
Field-collected samples	This study did not involve sample collected from the field.
Ethics oversight	All animal experiments of this study were conducted following the ARVO Statement for the Use of Animals in Ophthalmic and Vision Research and received approval from the Ethics Committee of animal experiments at the Zhongshan Ophthalmic Center (Guangzhou, China, acceptance number: 2019-044).

Note that full information on the approval of the study protocol must also be provided in the manuscript.
